# Residual Structures, Conformational Fluctuations, and Electrostatic Interactions in the Synergistic Folding of Two Intrinsically Disordered Proteins

**DOI:** 10.1371/journal.pcbi.1002353

**Published:** 2012-01-12

**Authors:** Weihong Zhang, Debabani Ganguly, Jianhan Chen

**Affiliations:** Department of Biochemistry, Kansas State University, Manhattan, Kansas, United States of America; Harvard University, United States of America

## Abstract

To understand the interplay of residual structures and conformational fluctuations in the interaction of intrinsically disordered proteins (IDPs), we first combined implicit solvent and replica exchange sampling to calculate atomistic disordered ensembles of the nuclear co-activator binding domain (NCBD) of transcription coactivator CBP and the activation domain of the p160 steroid receptor coactivator ACTR. The calculated ensembles are in quantitative agreement with NMR-derived residue helicity and recapitulate the experimental observation that, while free ACTR largely lacks residual secondary structures, free NCBD is a molten globule with a helical content similar to that in the folded complex. Detailed conformational analysis reveals that free NCBD has an inherent ability to substantially sample all the helix configurations that have been previously observed either unbound or in complexes. Intriguingly, further high-temperature unbinding and unfolding simulations in implicit and explicit solvents emphasize the importance of conformational fluctuations in synergistic folding of NCBD with ACTR. A balance between preformed elements and conformational fluctuations appears necessary to allow NCBD to interact with different targets and fold into alternative conformations. Together with previous topology-based modeling and existing experimental data, the current simulations strongly support an “extended conformational selection” synergistic folding mechanism that involves a key intermediate state stabilized by interaction between the C-terminal helices of NCBD and ACTR. In addition, the atomistic simulations reveal the role of long-range as well as short-range electrostatic interactions in cooperating with readily fluctuating residual structures, which might enhance the encounter rate and promote efficient folding upon encounter for facile binding and folding interactions of IDPs. Thus, the current study not only provides a consistent mechanistic understanding of the NCBD/ACTR interaction, but also helps establish a multi-scale molecular modeling framework for understanding the structure, interaction, and regulation of IDPs in general.

## Author Summary

Intrinsically disordered proteins (IDPs) are now widely recognized to play fundamental roles in biology and to be frequently associated with human diseases. Although the potential advantages of intrinsic disorder in cellular signaling and regulation have been widely discussed, the physical basis for these proposed phenomena remains sketchy at best. An integration of multi-scale molecular modeling and experimental characterization is necessary to uncover the molecular principles that govern the structure, interaction, and regulation of IDPs. In this work, we characterize the conformational properties of two IDPs involved in transcription regulation at the atomistic level and further examine the roles of these properties in their coupled binding and folding interactions. Our simulations suggest interplay among residual structures, conformational fluctuations, and electrostatic interactions that allows efficient synergistic folding of these two IDPs. In particular, we propose that electrostatic interactions might play an important role in facilitating rapid folding and binding recognition of IDPs, by enhancing the encounter rate and promoting efficient folding upon encounter.

## Introduction

It is now widely recognized that many functional proteins lack stable tertiary structures under physiological conditions [1]–[5]. Importantly, such intrinsically disordered proteins (IDPs) are highly prevalent in proteomes [6], play crucial roles in cellular areas such as signaling and regulation [7], [8], and are often associated with human diseases such as cancers [9]–[11]. The concept that intrinsic disorder can confer functional advantages has been discussed extensively [12]–[16]. For example, the disordered nature of IDPs could offer several unique benefits for signaling and regulation, including high specificity/low affinity binding, inducibility by posttranslational modifications, and structural plasticity for binding multiple partners. The last property appears to be particularly advantageous, and could support one-to-many and many-to-one signaling [16], [17]. Nonetheless, the physical basis of these proposed phenomena remains largely elusive. Specifically, how IDP recognition and regulation are supported by the interplay of residual structures, conformational fluctuations and other physical properties as encoded in the peptide sequence is poorly understood.

The current limit in mechanistic understanding of how intrinsic disorder supports function might be attributed to two key challenges in characterizing IDPs. These challenges are broadly shared by mechanistic studies of protein folding, misfolding, and aggregation in general [18]–[21]. The first one is related to the difficulty in deriving detailed structural information of the disordered unbound states [22]–[25]. In general, only ensemble-averaged properties can be measured for disordered proteins except with single-molecule techniques (which have their own limitations in spatial resolution, labeling need, and protein size [26]–[28]). Recovering the underlying structural heterogeneity using averaged properties is a severely underdetermined problem [29]–[33]. It is generally not feasible to construct a unique disordered structure ensemble that is consistent with the available data. This fundamental limitation leads to significant ambiguity in the current knowledge of the conformational nature of unbound IDPs. The second challenge is to further clarify the functional roles of any putative conformational sub-states or other properties of an IDP in its recognition and regulation (i.e., “function”). In particular, whereas some IDPs remain disordered in complexes [34], [35], many fold into stable structures upon binding to specific targets [36]. The roles of intrinsic disorder vs. residual structures in such coupled binding and folding interactions have been under much debate [36]. On one hand, residual structures have been observed frequently in unbound IDPs, and intriguingly, such residual structures often resemble those in the folded complexes [37]–[41]. These observations have led to an attractive hypothesis that preformed structural elements might provide initial binding sites to facilitate efficient recognition (i.e., conformational selection-like mechanisms) [12], [37]. On the other hand, evidence has accumulated in recent years, from computation as well as experimentation, to support a central role of nonspecific binding and emphasize the importance of disordered nature itself in promoting facile IDP recognition [41]–[51]. In fact, all published studies that extend beyond examining the unbound states alone have suggested induced folding-like mechanisms, at least at the baseline level.

Precisely how the disordered nature contributes to binding, however, is less clear. One proposal is that nonspecific binding of unstructured and presumably more extended conformations can increase the capture radii to enhance the binding kinetics [52], [53]; however, such “fly-casting” effects is small with a theoretical maximum of ∼1.6-fold acceleration. Recent studies have shown that unbound IDPs tend to be much more compact than previously assumed [54]–[57], further reducing the proposed fly-casting affects. In addition, the rate-enhancing affect due to increased size is likely offset by slower diffusion [58]. Alternatively, the unbound state of IDPs is presumed heterogeneous and strongly fluctuating. More specifically, conformational sub-states in the unbound IDPs should be marginally stable and separated by small free energy barriers (e.g., a few kcal/mol or less). These conformational fluctuations could contribute to efficient IDP recognition by allowing the peptide to fold rapidly upon (nonspecific) binding [50], [58], which is required for achieving the diffusion-controlled maximum binding rate (otherwise folding becomes rate-limiting) [59]. It should be noted that cellular events frequently modify the folding of IDPs to modulate their activities, such as through phosphorylations or by binding of other proteins [60]. Therefore, in contrast to globular proteins where folding often serves only to achieve the native structures, folding and unfolding appears to be direct and inherent aspects of IDP function. This underpins the importance and biological relevance of obtaining a mechanistic understanding of binding-induced folding of IDPs beyond a subject of theoretical curiosity.

The challenge in detailed characterization of IDPs represents a unique opportunity for molecular modeling to make critical contributions [5]. In particular, atomistic simulations could provide the ultimate level of detail necessary for understanding the structure and interaction of IDPs. At the same time, the dynamic and heterogeneous nature of IDPs also pushes the limits of both the force field accuracy and conformational sampling capability. So-called implicit solvent is arguably an optimal choice for *de novo* simulations of IDPs because of its necessary balance of accuracy and speed [61]–[64]. The basic idea of implicit solvent is to capture the mean influence of water by direct estimation of the solvation free energy, therefore reducing the system size about 10-fold. Important advances have been made to greatly improve the efficiency and achievable accuracy of implicit solvent, such as via the popular generalized Born (GB) theory [64]. With reduced system size, implicit solvent is also particularly suitable for replica exchange (REX) simulations [65]–[67], an enhanced sampling technique that has proven highly effective in sampling protein conformational equilibria [68]. Importantly, improved efficiency with implicit solvent also allows careful optimization to suppress certain systematic biases that have plagued explicit solvent approaches [69], [70]. For example, we have previously optimized the generalized Born with smooth switching (GBSW) model [71], [72] together with the underlying CHARMM22/CMAP protein force field [73]–[76]. The resulting GBSW protein force field not only recapitulates the structures and stabilities of helical and β-hairpin model peptides with a wide range of stabilities [77], [78], but also allows calculation of the conformational equilibria of small proteins under stabilizing and destabilizing conditions [79]–[81]. Although inherent and methodological limitations remain in implicit solvent [82], initial applications of implicit solvent to modeling small IDPs have been reasonably successful [41], [46], [55], [83]–[86], substantiating the notion that it is a viable approach for atomistic simulations of IDPs.

**Figure 1:**
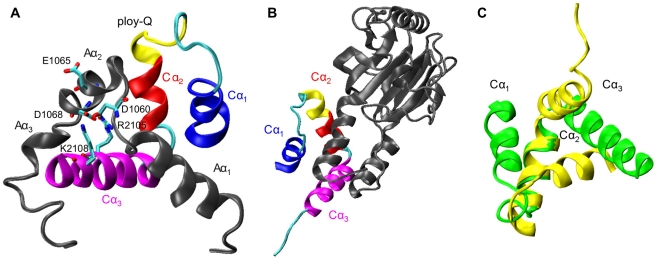
Two representative folded conformations of NCBD. A) NMR structure of the NCBD/ACTR complex (PDB: 1kbh [91], model 1). Both NCBD and ACTR contain 3 helical structure segments, labeled as Cα_1_ (blue), Cα_2_ (red) and Cα_3_ (magenta) in NCBD and Aα_1_, Aα_2_ and Aα_3_ in ACTR. See Methods for specific residue ranges of these helical segments. Several key structural features are also marked, including the poly-Q loop (yellow) linking Cα_1_ and Cα_2_, a buried salt-bridge between NCBD R2105 and ACTR D1068, and several key charged residues adjacent to this buried salt-bridge. B) X-ray crystal structure of the NCBD/IRF3 complex (PDB: 1zoq [94]). C) Overlay of the folded structures of NCBD in complex with IRF3 (yellow) and ACTR (green). Only the structured segment (residues 2066–2112) is shown, and the two structures are aligned using the backbone atoms of Cα_2_.

The current work focuses on the nuclear-receptor co-activator binding domain (NCBD) of the transcription coactivator CREB-binding protein (CBP) and its interaction with the p160 steroid receptor co-activator ACTR. CBP and its paralogue p300 are general transcriptional coactivators that play critical roles in transcriptional regulation and participate in cell cycle control, differentiation, transformation, and apoptosis [87], [88]. The NCBD domain (residues 2059–2117 in mouse CBP) is also known as interferon regulatory factor (IRF) binding domain (iBID) or the SRC1 interaction domain (SID). It mediates the interaction of CBP with a number of important proteins, including steroid receptor coactivators, p53 and IRFs [2], [89]. The interaction of CBP with p160 coactivators in particular is important for recruitment of CBP/p300 to transmit the hormonal signal to the transcription machinery [90]. Besides the biological and medical significance, the NCBD/ACTR interaction also offers unique opportunities for understanding the molecular principles of IDP recognition. Both NCBD and the activation domain of ACTR that it interacts with (residues 1018–1088 in human ACTR; hereafter referred to as ACTR) are IDPs. Their interaction is an example of the “synergistic folding” mechanism [91] (the other known example also involves NCBD, but with the p53 transactivation domain, TAD [92]). In addition, four folded structures of NCBD have been solved in complex with various protein targets besides ACTR [91]–[94]. In these complexes, NCBD adopts two distinct tertiary folds that involve three similar helices, represented by the NCBD/ACTR and NCBD/IRF3 complexes (see Figure 1). Therefore, NCBD represents one of the few experimentally validated examples of structural plasticity, which is believed to be a key functional advantage of intrinsic disorder [16].

Interestingly, although free ACTR is largely devoid of residual structures, free NCBD contains one the highest levels of residual structures with folded-like helical content and molten globule characteristics [95], [96]. In addition, even though nuclear magnetic resonance (NMR) relaxation analysis has established that free NCBD is highly dynamic on picosecond (ps) to nanosecond (ns) timescales [96], it appears to have a strong tendency to adopt marginally stable tertiary folds, allowing two NMR structures of the unbound state determined to date [40], [89]. These structures are presumably obtained by stabilizing various conformational sub-states under specific solution conditions. Particularly intriguing is that the latest NMR structure of free NCBD turns out to be similar to the folded conformation observed when bound to ACTR [40]. Although such pre-existence of folded-like conformations should be considered only as a necessary but insufficient condition for conformational selection-like mechanisms, the unusually high level of residual structures of NCBD strongly suggests a functional role of pre-folding in its coupled binding and folding interactions. In this work, we first exploit implicit solvent-based atomistic simulations and REX enhanced sampling to characterize the conformational properties of free NCBD and ACTR. The roles of preformed structures vs. conformational fluctuation in the NCBD/ACTR interaction are then directly probed using high-temperature unfolding and unbinding simulations in both implicit and explicit solvents. Combined with our recent coarse-grained simulations and existing experimental data, we aim to obtain a detailed mechanistic picture of how residual structures, conformational fluctuations, and electrostatic interactions contribute to efficient synergistic folding of NCBD and ACTR.

## Results

### Convergence and validation of the disordered structure ensembles

*De novo* calculation of the disordered ensembles for IDPs is challenging [5], especially for NCBD that is both of moderate size and apparently with a complex, solution condition-sensitive conformational equilibrium. Our previous works have suggested that implicit solvent coupled with REX enhanced sampling could generate reasonably accurate disordered ensembles for small IDPs, including a 28-residue segment of the kinase inducible domain (KID) of transcription factor CREB [55]. In Figure S1, we first test the convergence of the calculated disordered ensembles by examining the dependence of residue helicity on REX simulation time and by comparing results from independent simulations initiated from dramatically different conformations (folding vs. control; see Methods). The sequences of both domains are provided in Methods. Free ACTR appears to be highly disordered with marginal residual helicity. The calculated residual helicity profiles from the control and folding runs converge to similar ones (data not shown). For NCBD, while the time evolution of the calculated residual helicity appears to stabilize over the course of 100 ns in either the control or folding REX simulation, the final profiles from these two independent calculations differ substantially, suggesting that the actual convergence is rather limited. Nonetheless, both the folding and control simulations clearly suggest significant residual helicity in all three helical segments that become stably folded upon binding to various specific targets. Detailed analysis of the conformational ensemble (see below) demonstrates that free NCBD is compact and contains substantial tertiary contacts. These conformational properties of NCBD, coupled with the larger size, contribute to the difficulty of achieving better convergence using the REX/GB protocol. In addition, the current surface area-based treatment of nonpolar solvation can over-stabilize non-specific collapsed states [82], [97]. This problem further limits the ability to sufficiently sample accessible tertiary organizations of free NCBD and their inter-conversions, which is required for achieving good convergence.

**Figure 2:**
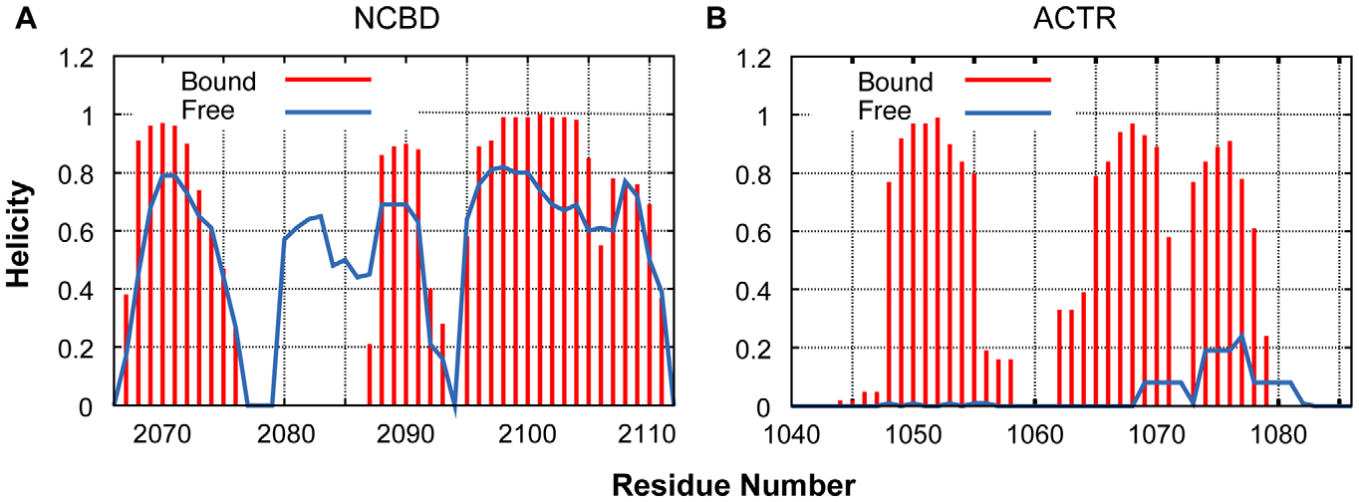
Calculated residue helicity of NCBD and ACTR in the free and bound states. Results for the bound state were calculated from a 100 ns control simulation of the complex (see Methods), and those for the free peptides were calculated based the conformationals sampled at 305 K during the last 60 ns of the control REX simulations.

Given the limited convergence achieved in the REX simulations of free NCBD and apparent difficulties in substantially improving the level of convergence, we focus on semi-quantitative or qualitative analysis of the conformational properties of NCBD. That is, although significant conformational sub-states sampled by REX may be genuine, the relative stability (population) is not likely to be reliable. Considering that NCBD is experimentally known to be highly helical, the folding simulations (initiated with a fully extended conformation) should take longer to converge, and the disordered ensemble calculated from the control simulation is likely more realistic. Therefore, all the subsequent analysis is based on the ensemble of conformations sampled during the last 60 ns of the 100 ns control REX simulation. In Figure 2, we compare the residue helicity of NCBD and ACTR in the free and bound states. The results appear to be fully consistent with the previous NMR secondary chemical shift analysis (Figure 2 of Ref. [96]), showing that all three NCBD helices are largely formed in the unbound state and ACTR is largely free of residual helices. Interestingly, the poly-Q segment of NCBD (residues 2082–2086), although disordered in the NCBD/ACTR complex, is largely helical in the unbound state and extends Cα_2_. This is fully consistent in the NMR chemical shift analysis [96]. Recent sequence correlation analysis has revealed a link between sequence order and binding promiscuity [98], [99]. One might expect that the length of the poly-Q stretch might affect conformational flexibility, and furthermore, the ability to interact with diverse targets. We also have analyzed the ensemble distribution of the radius of gyration of free NCBD. The results, shown in Figure S2A, confirm that free NCBD is highly compact. Despite a clear lack of convergence, the control and folding simulations appear to sample a set of conformation sub-states with similar characteristic sizes. Direct comparison of the calculated size profiles to one derived from a recent small-angle X-ray scattering (SAXS) study [40] is complicated by the different constructs used and uncertainty in proper inclusion of the solvation shell for a heterogeneous ensemble. Nonetheless, one can estimate that including the disordered N- and C-terminal tails (13 residues total) truncated in the current simulations would increase the radius by 2–3 Å, and that the solvation shell may add another 2–3 Å (estimated by comparing results from HydroPro [100] and CHARMM). These corrections together bring the calculated radius of the gyration profile close to the SAXS-derived profile that centers around 15.2 Å under “native-like” conditions [40]. Apparent agreement between NMR and SAXS on these ensemble-averaged properties is not sufficient to validate the reliability of the simulations, but it suggests that the simulated ensemble may offer a qualitative or even semi-quantitative characterization of the conformational properties of free NCBD.

### Folded-like conformations in the unbound state of NCBD

**Figure 3:**
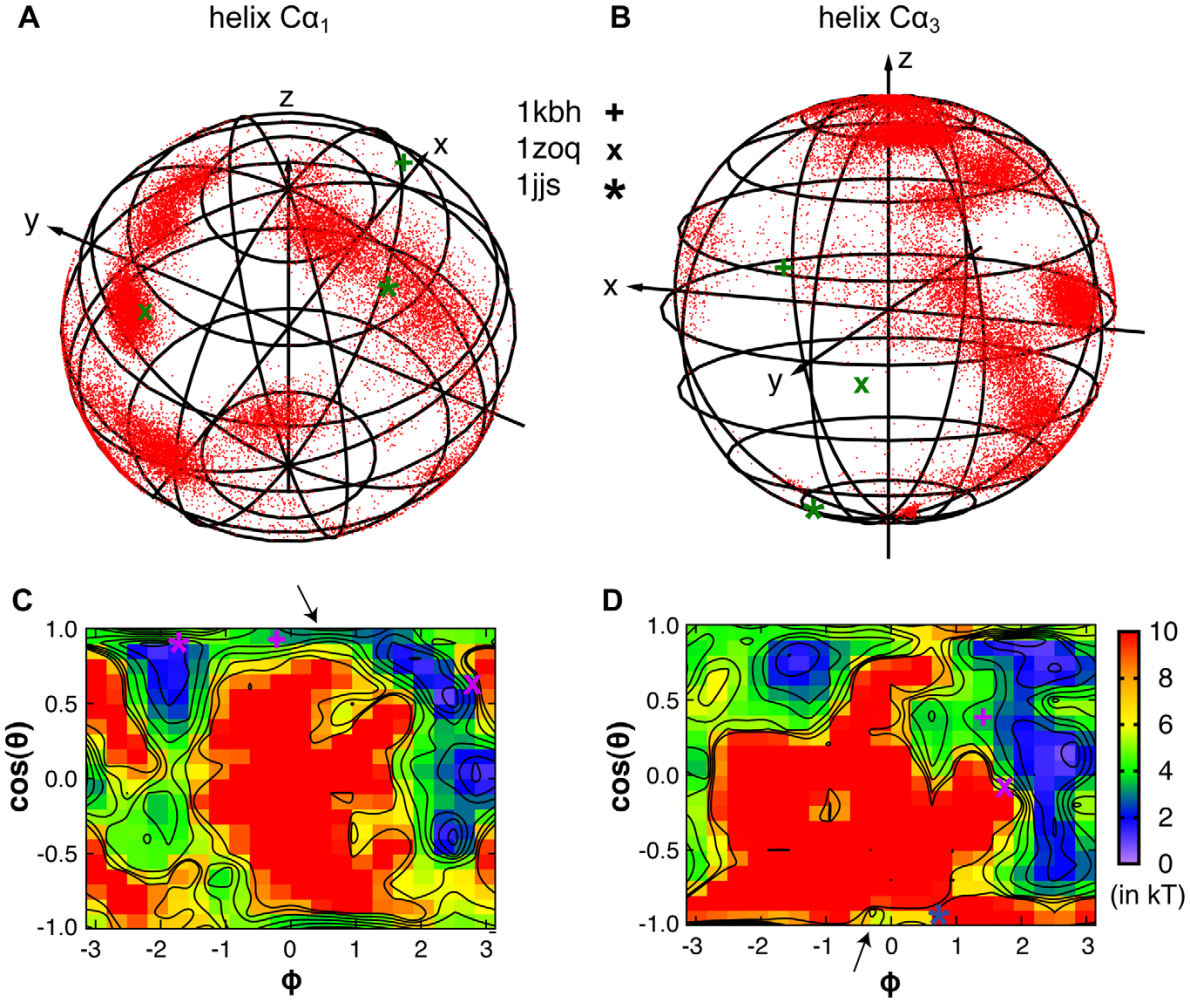
Orientations of NCBD Cα_1_ and Cα_3_ with respect to Cα_2_ in the unbound state. All conformations sampled at 305 K during the last 60 ns of the control REX simulation were first aligned using the backbone atoms of Cα_2_, and then reoriented such that Cα_2_ was aligned with −z axis. The orientations as observed in three distinct folds of NCBD, represented by PDB 1kbh, 1zoq, and 1jj (also see Figure 1), are marked with “+”, “×”, and “★”, respectively. Note that different colors for the same symbol may be used in different panels for clarity. In panels c) and d), ϕ and θ are the inclination and azimuth angles of the spherical coordinate system. Note that the PMFs were computed using sin(θ) instead of θ itself as an order parameter to remove the Jacobian entropy contribution. The range shown corresponds to θ = 0 (top) to π (bottom). Contours are drawn at every kT up to 7 kT, with k being the Boltzmann factor.

Because all three NCBD helices are largely formed in the unbound state, the conformational fluctuation of free NCBD mainly involves tertiary packing of these helices. For example, as shown in Figure 1, when aligned using the central helix Cα_2_, the two representative folded conformations of NCBD differ mainly in the orientation of Cα_1_ and slightly less so in that of Cα_3_. Therefore, all conformations of the calculated ensemble first re-oriented by aligning Cα_2_ (to the −z axis) before the orientations of Cα_1_ and Cα_3_ were calculated. Note such analysis also provides an effective description of the tertiary packing even when one or more of the three NCBD segments are not in helical states. The results, shown in Figure 3, illustrate that NCBD is strongly fluctuating and samples a large number of helix configurations, as expected for a molten globule. Intriguingly, free NCBD appears to substantially sample all three distinct conformations that have been observed experimentally so far, either in complexes or in isolation. These folds are represented by PDB structures 1kbh, 1zoq, and 1jjs, respectively. The Cα_1_ orientation of 1kbh and Cα_3_ orientation of 1jjs appear to be least sampled. Nonetheless, conformational sub-states exist with similar orientations, as marked by arrows in panels c) and d) of Figures 3. Specifically, for 1kbh-like Cα_1_ orientation, the adjacent sub-state contains more parallel (with smaller helix cross angles), and thus tighter, packing of Cα_1_ with Cα_2_, but with a helix interface similar to that of 1kbh. Further structural analysis (see the following paragraph) suggests that such tighter packing is likely a result of helix formation in the poly-Q segment (e.g., see Figure 2), which shortens the Cα_1_-Cα_2_ loop and promotes tighter packing.

**Figure 4:**
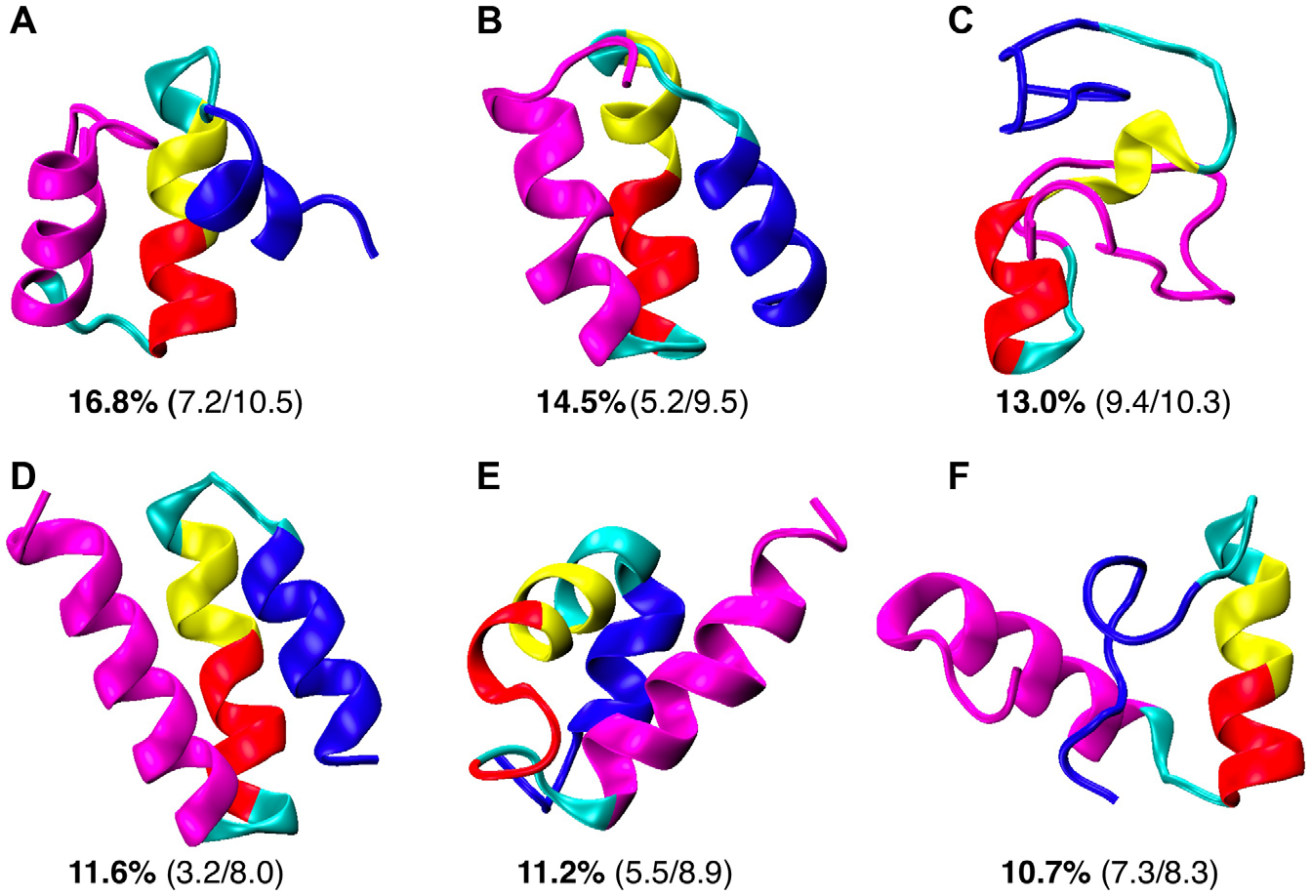
Averaged structures of the six most populated clusters of free NCBD. These clusters are identified based on the conformations sampled at 305 K during the last 60 ns of the 100 ns REX control simulation. All structures were aligned by minimizing the backbone RMSD of Cα_2_ (the red segments) and visualized in the same view. The numbers below each structure are the population of the cluster and backbone RMSD values from the folded conformations in 1kbh and 1zoq (see Figure 1). The protein is colored in the same fashion as in Figure 1a–b.

Clustering analysis was performed to further analyze the structural properties of the major conformational sub-states of free NCBD. The average structures of the six most populated clusters identified using K-means clustering with a 3.0 Å radius are shown in Figure 4. Helix configurations for all members of these clusters are shown in Figure S3. Interestingly, even though one of the clusters (Figure 4D) is similar to the fold observed in 1kbh, most clusters are different from either 1zoq or 1kbh on the whole domain level, as suggested by the large RMSD values. Therefore, even though both individual Cα_1_-Cα_2_ and Cα_2_-Cα_3_ helix pairs sample all three distinct PDB folds, these folded-like configurations of individual helix pair generally do not occur at the same time. Notably, the folded conformations of NCBD in 1kbh and 1zoq have relatively similar Cα_2_-Cα_3_ helix packing (see Figure 1C). The packing of Cα_2_ and Cα_3_ also appears to be more restricted in free NCBD compared to that of Cα_1_ and Cα_2_ (e.g., as indicated by a larger “inhibited” red area in Figure 3D compared to Figure 3C). NCBD has a strong inherent propensity to adopt Cα_2_-Cα_3_ configurations analogous to those in 1kbh and 1zoq. Such persistent folded-like conformations of free NCBD could contribute to recruitment of specific targets such as ACTR and IRF3, allowing NCBD to adopt different final structures by docking the more flexibly linked Cα_1_ into different positions. Another interesting observation is that the poly-Q segment appears to be capable of readily switching between helical and coil states. Such conformational fluctuations could allow NCBD to adapt to different substrates, extending the Cα_2_ helix when bound with IRF3 but becoming more disordered when in complex with ACTR (see Figure 1).

### Induced folding-like mechanism on the baseline level

Although the REX simulations provide intriguing insights into the possible residual structures of free NCBD, how these conformational properties contribute to synergistic folding of NCBD with ACTR is not obvious based on these equilibrium simulations alone. For this, one could calculate the coupled binding and folding free energy surfaces [41], [51] or transition paths [46] to more directly clarify the recognition mechanism and probe the roles of residual structures vs. pre-folding in specific finding. However, given the moderate size and relatively complex topology, such calculations can be extremely demanding using an atomistic physics-based force field for the NCBD/ACTR complex. Instead, temperature-induced unfolding and unbinding simulations may be used to effectively *infer* the molecular processes of coupled binding and folding. A key assumption is that binding/folding is largely a reverse of unbinding/unfolding. An important concern is that the transition states or the most probable transition paths might depend on temperature [101]. Nonetheless, high-temperature unfolding simulations have so far proven quite successful for studying folding and interaction of many proteins, including IDPs [44], [102]–[104].

**Figure 5:**
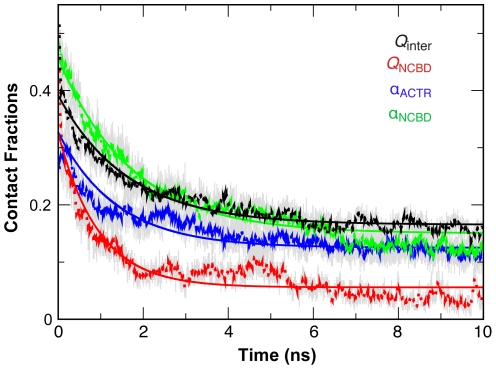
Evolutions of various contact fractions of the NCBD/ACTR complex at 475 K. All contact factions and helicities were computed by averaging results from 50 independent unfolding simulations. *Q*_inter_ and *Q*_NCBD_ denote the native fractions of intermolecular and NCBD tertiary intramolecular contacts, and *α*_ACTR_ and *α*_NCBD_ denote the overall helicities of ACTR and NCBD. Significant fluctuation remains in the raw averaged contact fraction traces (grey traces), and thus 50-ps running averages (dotted traces) are plotted for clarity. The solid traces correspond to the best single exponential fits (see Table 1 for the fitted kinetic constants). Note that both *Q*_inter_ and *Q*_NCBD_ quickly decrease from above 0.8 to ∼0.4 during the first 10–20 ps. The initial decay is out of the plotting range and not shown for clarity.

A 100 ns equilibrium simulation of the complex was first performed at 300 K, which confirms that the native fold (model 1 of PDB:1kbh) is very stable in the GBSW/MS2 implicit solvent (see Figure S4). Subsequent pilot simulations suggest 475 K to be optimal for simulating unbinding and unfolding of the NCBD/ACTR complex in GBSW/MS2 (e.g., see Figure S5). In Figure 5, we compare the time evolution of various fractions of native contacts computed from 50 independent unfolding simulations at 475 K. The fraction of native intermolecular interactions (*Q*_inter_) is used to describe binding, and the fraction of native tertiary intramolecular interactions (*Q*_NCBD_) is used for folding of NCBD. As shown in Figure S6, ACTR is completely devoid of any inter-helix tertiary contacts in the NCBD/ACTR complex. Because ACTR is largely free of residual structures in the unbound state, the overall helicity (α_ACTR_) is used to effectively monitor (binding-induced) folding of ACTR. On the baseline level, all unfolding and unbinding kinetics appear to be reasonably well represented by single exponential functions. The fitted kinetic data is summarized in Table 1. The secondary (helix) unfolding of NCBD is predicted to be the slowest process (α_NCBD_; green traces in Figure 5), which is expected given the high level of residual structures in unbound NCBD; however, both the ACTR (helix) and NCBD tertiary unfolding appear to be significantly faster than unbinding. This result suggests that binding occurs prior to the folding of both ACTR and NCBD; that is, both ACTR and NCBD follow induced-folding-like mechanisms on the baseline level in the GBSW/MS2 implicit solvent. Considering the apparent tendency of NCBD to pre-fold (see above), this result is somewhat surprising, but it highlights the importance of conformational fluctuations and nonspecific binding in specific recognition of IDPs, even for IDPs with significant residual structures like NCBD. Significant heterogeneity is apparent in the unfolding/unbinding pathways of NCBD/ACTR and is partially reflected in substantial ruggedness that remains in the curves shown in Figure 5 (e.g., compared with a previous explicit solvent unfolding simulation of the p53-MDM2 complex, where 10 10-ns simulations at 498 K were sufficient to yield much smoother curves [103]). The complex fully disassociates within 10 ns in only 6 out of the 50 independent runs. In examining the unbinding/unfolding characteristics at a lower temperature of 450 K (see Figure S7), we found the heterogeneity of unfolding/unbinding pathways to be even more evident. In addition, the complex appears trapped in some intermediate states and does not fully unfold/unbind even after 20 ns. Nonetheless, unfolding of either ACTR or NCBD appears to lag behind unbinding, which is consistent with the induced-folding baseline mechanisms predicted at 475 K.

**Table 1:** Unfolding and unbinding kinetic constants at 475 K.

	τ (ns)	*A*	*B*	*R* ^2^
*Q*_inter_	1.61	0.23	0.17	0.94
Cα_1_	0.26	0.37	0.00	0.97
Cα_2_	1.52	0.25	0.034	0.84
Cα_3_	2.20	0.24	0.23	0.94
Aα_1_	0.80	0.34	0.037	0.94
Aα_2_	1.39	0.26	0.26	0.86
Aα_3_	2.93	0.32	0.30	0.86
*Q*_NCBD_	0.94	0.27	0.055	0.90
α_NCBD_	1.76	0.31	0.15	0.96
α_ACTR_	1.38	0.20	0.13	0.89

All curves were fitted by single exponentials *A* exp (−t/τ)+*B*. *R* is the correlation coefficient of fitting. See the captions of Figures 5 and 7 for the definitions of various contact fractions.

### Binding and folding intermediates involving the NCBD and ACTR C-terminal segments

**Figure 6:**
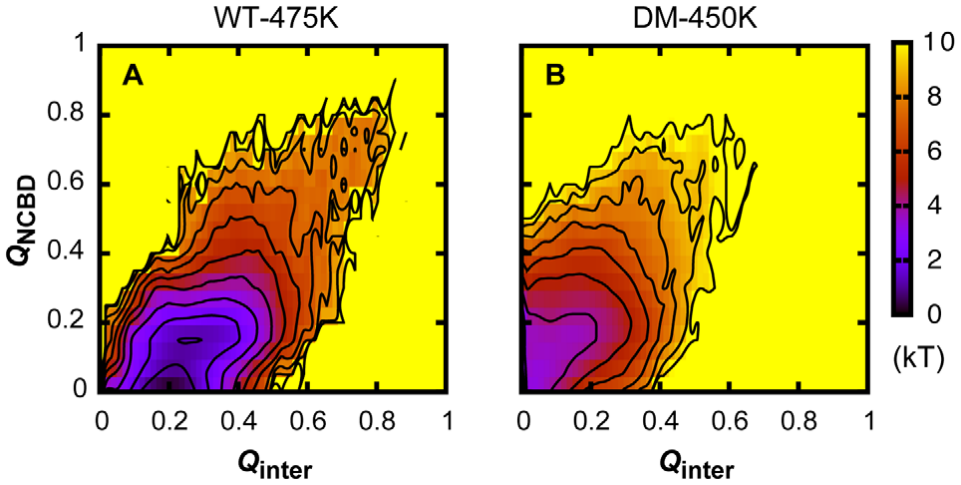
Free energy surfaces of (un)binding and (un)folding of the wild-type and mutant NCBD/ACTR complex. A. Computed from the first 5 ns of 50 independent simulations of the wild-type NCBD/ACTR complex (WT) at 475 K. B. Computed from the first 5 ns of 50 independent simulations of the double-Leu mutant complex (NCBD:R2105L/ACTR:D1068L; DM) at 450 K. Contours are drawn at every kT.

Indications are that binding-induced folding of NCBD and ACTR is not simply 2-state-like. For example, decay of *Q*_NCBD_ and *α*_ACTR_ appears to pause at ∼2 ns (red and blue traces in Figure 5), which could suggest a common intermediate state where ACTR and NCBD are partially bound and folded. The decay curves are too noisy (partially due to underlying heterogeneity) for reliable kinetic fitting using double exponential functions. Therefore, we constructed (pseudo) unbinding and unfolding free energy surfaces based on statistics collected from the first 5 ns of the unfolding simulations. Note that the system is not at equilibrium during this time frame, so the resulting free energy profiles are not equilibrated (and thus strongly dependent on initial conditions). Nonetheless, the profiles provide qualitative approximations of the true free energy surfaces [105]. As shown in Figure 6A, an intermediate state is evident at *Q*_inter_∼0.25 and *Q*_NCBD_∼0.15. Interestingly, a similar key intermediate state also has been predicted in our recent topology-based modeling of the NCBD/ACTR complex [50]. A strong resemblance between the free energy surface is shown in Figure 6A and the result derived from topology-based modeling (Figure 5A of reference [50]). Both the atomistic simulations (see further analysis detailed in the following paragraph) and topology-based modeling predict that the intermediate state mainly involves the C-terminal segments of NCBD and ACTR. Such a prediction appears highly consistent with a recent H/D exchange mass spectrometry (H/D-MS) study [106], where peptide segments within the C-terminal regions of both NCBD and ACTR were found to have much larger protection factors compared with those mapped into other folded regions of the complex.

**Figure 7:**
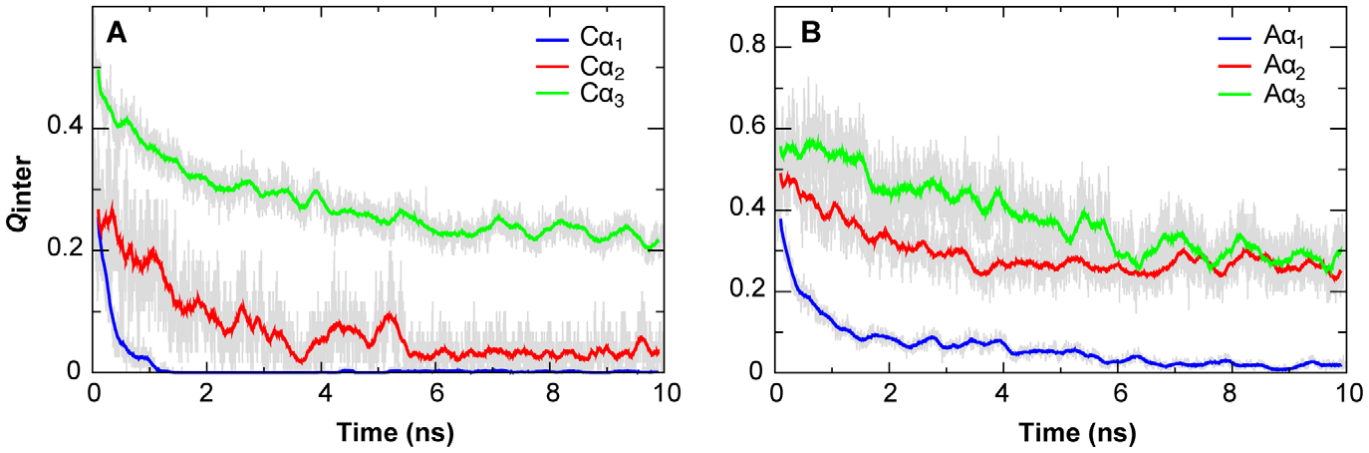
Evolutions of the fractions of native intermolecular interactions of individual helices of the NCBD/ACTR complex. The grey traces were calculated from averaging 50 unfolding simulations at 475 K and the colored traces are 50-ps running averages.

In Figure 7, we further examined the binding kinetics of individual NCBD and ACTR helices. The kinetic data derived from fitting to single exponential functions is summarized in Table 1. The analysis shows that Aα_3_ and Cα_3_ unbind with the largest half times, τ = 2.93 ns and 2.20 ns, respectively, which are greater than that of the overall intermolecular interaction formation (τ = 1.61 ns). This result indicates that binding is mainly initiated by the C-terminal helices. In contrast, the first helices of NCBD and ACTR unbind much faster then the second and third helices. In fact, unbinding of Aα_1_ and Cα_1_ occurs even faster than folding of either NCBD or ACTR (as described by *Q*_NCBD_ and α_ACTR_, see Table 1). These kinetic rates are consistent with a multi-stage synergistic folding process, where NCBD and ACTR first bind rapidly through the C-terminal segments, forming intermediates that are mainly stabilized by native-like interactions between α_2_ and α_3_ helices. This first step appears to be highly cooperative (e.g., see Figure 6A), although indications are that both induced folding and conformational selection might contribute [50]. Interestingly, the transition between the intermediate and bound states appears largely conformational selection-like where NCBD and ACTR folding precedes Aα_1_ and Cα_1_ binding. Formation of the partially folded core appears to facilitate the rest of NCBD to fold into native-like conformations, allowing Cα_1_ and Aα_1_ to rapidly form native intermolecular interactions en route to the fully folded bound state. Taken together, even though the synergistic folding of NCBD and ACTR follows an induced folding-like baseline mechanism (where binding precedes folding on the overall level), detailed analysis reveals multiple stages of induced folding and conformational selection. Such a mechanism closely resembles an “extended conformational selection” recently proposed by Csermely et al. [107], [108] and is remarkably consistent with our recent topology-based modeling of the NCBD/ACTR complex [50].

### Native and non-native salt-bridges in encounter complexes and intermediates

**Figure 8:**
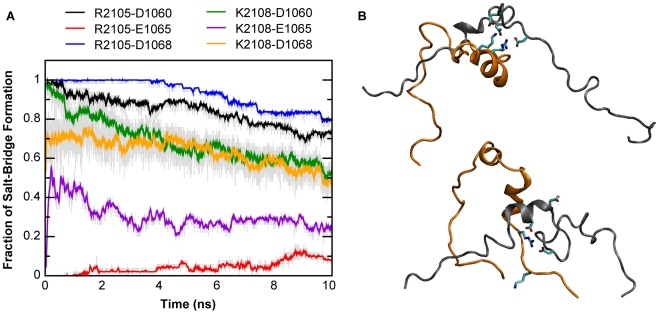
Native and non-native salt-bridges in the NCBD/ACTR interaction. A. Evolutions of average probabilities of various salt-bridge interactions during unfolding simulations at 475 K. Arg and Glu/Asp residues were considered in contact if the carbonyl carbon and Arg C_Z_ distance was no greater than 5 Å, and Lys and Glu/Asp residues were considered in contact if the side chain carbonyl carbon and amide nitrogen distance is no greater than 4 Å. B. Two representative final conformations after 10 ns simulations at 475 K. NCBD and ACTR are colored orange and gray, respectively. The side chains of key charged residues are also shown, including NCBD R2105 and K2108 and ACTR D1060, E1065, and D1068. The snapshot on the top represents a case where all six possible salt-bridges are formed, and the one at the bottom represents a case where only the native ones, between NCBD R2105 and ACTR D1068 and D1060, are formed.

One of the most notable features of the NCBD/ACTR complex is a buried salt-bridge between NCBD R2105 and ACTR D1068 [91] (see Figure 1A), which is also conserved in the interaction of NCBD with p53 TAD [92]. Interestingly, this buried salt-bridge is part of a local network of salt-bridges that could form between multiple complementary charges, including R2105 and K2108 of NCBD and D1060, E1065, and D1068 of ACTR (see Figure 1A). This network of native and non-native salt-bridges appears to play a significant role in stabilizing the putative intermediate state, either thermodynamically or kinetically. Although most individual salt-bridges frequently break and reform during individual unfolding simulations (see Figure S8), on average they largely persist throughout the 10 ns unfolding simulations at 475 K and hinder the transition from the partially bound intermediates to fully disassociated ones (see Figure 8). Out of the 50 unfolding simulations at 475 K, the complexes fully dissociate only by the end of 10 ns simulations in six cases. The native salt-bridges, between NCBD R2105 and ACTR D1068 and D1060, are the most protected. As shown in Figure 8, they are the most preserved and remain formed over 80% of the time throughout the simulations (blue and black traces in Figure 8A). NCBD K2108 is adjacent to R2015 and close enough to interact with ACTR D1068 and D1060, but these salt-bridges are more solvent-exposed and thus slightly less preserved during high-temperature simulations. The side chain of ACTR E1065 is positioned away from NCBD in the native structure. Partial unfolding of Aα_2_ allows E1065 to rotate and participate in the salt-bridge network with 10–30% probability by the end of the 10 ns simulation at 475 K (purple and red traces in Figure 8A).

**Figure 9:**
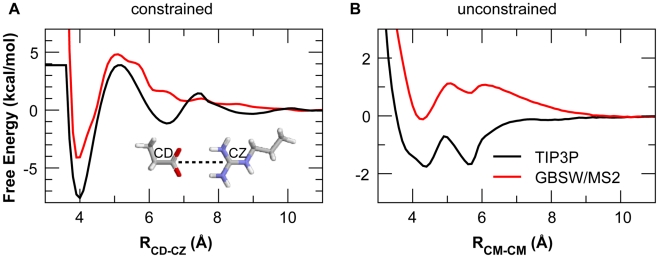
Potential of mean forces of the Arg-Asp interactions in implicit and explicit solvents. All profiles were calculated using umbrella sampling and WHAM (see Methods). The center-of-mass (CM) separation is used as the order parameter in the unconstrained PMF.

The conformational heterogeneity of the intermediate state does not permit reliable free energy calculations to quantify the contribution of salt-bridge interactions to stability. Nonetheless, previous mutagenesis studies have suggested that the buried salt-bridge between NCBD R2105 and ACTR D1068 contributes minimally to binding affinity [95]. The salt-bridge network likely could not significantly stabilize the intermediate state thermodynamically, either, which raises a concern that the observed persistence of the local salt-bridge network is artificial, such as due to over-stabilization of charge-charge interactions in the GBSW/MS2 implicit solvent. To address this concern, we first examine the potential of mean forces (PMFs) between Arg and Asp side chain analogs in TIP3P and GBSW/MS2. The results, summarized in Figure 8, show that GBSW/MS2 actually slightly under-stabilizes the Arg-Asp interaction compared with TIP3P, either in a constrained head-to-head configuration (which was used in the force field optimization [72]) or when fully unconstrained. In particular, configurationally unconstrained Arg-Asp interaction is unstable in GBSW/MS2 (Figure 9B). Therefore, the observed stabilization effects of salt-bridges on the intermediates are likely of a kinetic nature. Such kinetic stabilization arises from substantial desolvation barriers in disassociation of salt-bridges, particularly in partially folded protein environments where the side chain configurations are restricted (e.g., see Figure 9A). With a concentrated local network of salt-bridges, very large desovaltion barriers can be expected for complete dissociation of NCBD and ACTR, which explains why only a small fraction of the high-temperature simulations (6 out of 50) successfully reached the fully unbound state in 10 ns.

To further confirm that the observed salt-bridge network is not an artifact of implicit solvent, a set of 10 unfolding simulations was performed in TIP3P explicit solvent at 500 K. Most simulations were terminated between 3 to 4 ns when the complex size exceeded the periodic box dimensions. The lengths of these simulations are insufficient to capture degrees of unfolding and unbinding similar to implicit solvent simulations, and the number of trials is insufficient to obtain smooth curves for kinetic fitting. Nonetheless, visual inspection of simulation trajectories as well as examination of the evolution of various contact fractions support an unbinding and unfolding mechanism that is consistent with the one derived from implicit solvent simulations (see Figure S9). The same set of native and non-native interactions, particularly the buried one between NCBD R2105 and ACTR D1068 (blue trace in Figure S9B), persist and appear to stabilize the partially unbound and unfolded intermediates. Note that the helical secondary structures are substantially over-stabilized in these explicit solvent simulations (e.g., see the blue trace in Figure S9A). This is a known artifact of the current version CHARMM22/CMAP explicit solvent force field [78], [109], [110].

### A double-Leu mutant complex follows a similar unfolding and unbinding mechanism

A control simulation of the double-Leu mutant complex, NCBD:R2105L/ACTR: D1068L, at 300 K suggests that the native fold remains stable in the GBSW/MS2 implicit solvent (data not shown). A set of 50 unfolding simulations was carried out at 450 K to further investigate the role of the buried salt-bridge in synergistic folding. The heterogeneity of the unfolding/unbinding pathway observed in the wild-type complex (e.g., see Figure 5) is even more pronounced without the buried salt-bridge. All averaged time traces of contact fractions remain very noisy (e.g., see Figure S10). Most traces cannot be satisfactorily fitted to either single or double exponential functions, preventing quantitative analysis of unfolding and unbinding kinetics. Nonetheless, the pseudo binding and folding free energy surface computed from the first 5 ns of the unfolding trajectories appears to resemble that from simulations of the wild-type complex (see Figure 6). In particular, a similar intermediate state exists at *Q*_inter_∼0.2 and *Q*_NCBD_∼0.15; however, the small free energy barrier separating the intermediate and fully unbound states in Figure 6A is largely absent in Figure 6B. Removal of NCBD:R2105L largely disrupts the local salt-bridge network. The intermediate state appears to have much shorter resident times, and can quickly fluctuate to the fully unbound state. Importantly, examination of the evolution of intermolecular contact factions of individual NCBD and ACTR helices, shown in Figure S10, supports that the mutant complex largely follows a similar, albeit more heterogeneous, unbinding and unfolding mechanism, with the N-terminal α_1_ helices disassociated first (black traces in Figure S10B–C). These results suggest the local salt-bridge network does not appear to fundamentally modulate the recognition mechanism. Instead, it mainly augments a productive synergistic folding mechanism inherent in (the topology of) the NCBD/ACTR complex, by transiently stabilizing a key on-pathway intermediate state to facilitate complete folding en route to the specific complex.

## Discussion

**Figure 10:**
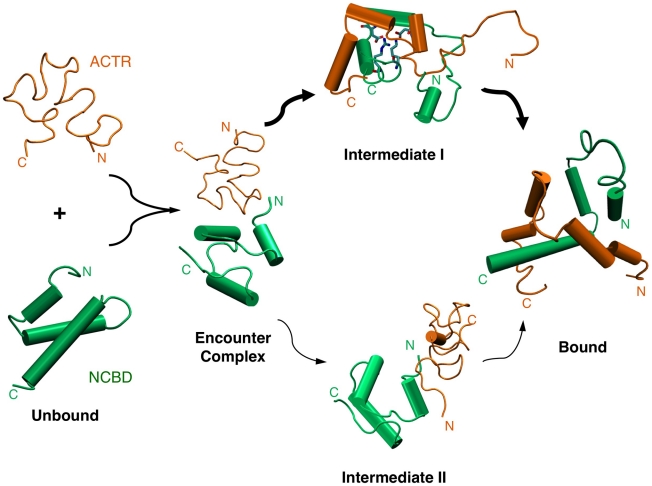
Overall mechanism of synergistic folding of NCBD and ACTR. The schematic view is based on the current atomistic simulations as well as the previous coarse-grained modeling [50]. It illustrates that unbound ACTR is largely unstructured and NCBD has significant helical structures. The nonspecific encounter complexes could evolve toward the bound state through two parallel pathways. The salt-bridge network that plays a key role in stabilizing Intermediate I along the prevalent pathway is also illustrated.

With one of the highest levels of residual structures, NCBD is an intriguing model system for understanding the roles of residual structure vs. conformational fluctuations in coupled binding and folding of IDPs. We have combined equilibrium and non-equilibrium simulations using physics-based, atomistic protein force fields to characterize the conformational properties of unbound NCBD and ACTR and to understand how these properties facilitate efficient synergistic folding of these two IDPs. The calculation recapitulates that free NCBD has folded-like helical content, is strongly fluctuating, and samples a wide range of tertiary configurations, which is consistent with the previous notion that free NCBD is a molten globule [96]. Intriguingly, the calculated disordered ensemble of NCBD contains significant populations with helical packings that are highly similar to all those previously observed experimentally in isolation and in complex with various targets. Observations of such pre-folded conformations, especially for IDPs with significant residual structures like NCBD, could be considered strong evidence for conformational selection-like mechanisms, where such preformed structural elements provide initial binding sites. Direct examination of the unfolding and unbinding pathways in high-temperature simulations, however, shows that both ACTR and NCBD tend to unfold first before unbinding, suggesting an induced folding-like baseline mechanism for their synergistic folding. This seemingly surprising result appears to be consistent with the observation that, although individual Cα_1_/Cα_2_ and Cα_2_/Cα_3_ helical pair samples folded-like packing with substantial probability, these configurations rarely occur simultaneously. Therefore, population of folded-like tertiary conformations on the whole domain level is insufficient to support conformational selection-like mechanisms on the baseline level. Further analysis reveals an on-pathway intermediate state that mainly involves the C-terminal helices of ACTR and NCBD, which also has been predicted by a recent coarse-grained simulation study using topology-based models [50]. Importantly, existence of such a major intermediate state also appears to be consistent with a recent H/D-MS experiments showing that peptide segments within the C-terminal regions of NCBD and ACTR have much larger protection factors compared with those mapped into other regions of the complex [106]. Our kinetic analysis suggests that, once the initial mini folding core is formed, the N-terminal helix of NCBD folds rapidly (Table 1), allowing subsequent facile binding and folding the ACTR N-terminal helix en route to the final specific complex. Therefore, although the baseline mechanism is induced folding-like, conformational selection actually occurs at local levels. Together with our recent topology-based modeling study [50], the atomistic simulations strongly support the prediction that synergistic folding of NCBD and ACTR follows the “extended conformational selection” mechanism [107]. Our topology-based modeling of the NCBD/ACTR interaction [50] has revealed a separate, albeit less prevalent, pathway where binding is initiated by the N-terminal α_1_ helices. These mechanistic insights on synergistic folding of NCBD and ACTR, derived from the atomistic and coarse-grained simulations, are summarized in Figure 10.

An intriguing interplay appears to exist among residual structures, conformational fluctuations, and electrostatic interactions to facilitate the rate-limiting step of forming the partially folded intermediates. The NCBD Cα_2_/Cα_3_ helix-turn-helix motif appear to be conformationally more restricted (Figure 2D), whereas the C-terminus of Cα_3_ retains the least amount of helical content and is considerably more heterogeneous (Figure S2B). Both features were also observed in the previous NMR chemical shift and relaxation analysis [96]. Such a balance of residual structures and conformational fluctuations is likely important for the NCBD C-terminal to act as a key initiation point for coupled folding and binding to ACTR and other proteins. Another novel insight provided by the current atomistic simulations is the role of a local network of native and non-native salt-bridges in transiently stabilizing the intermediates. These salt-bridge interactions likely do not contribute substantially to the thermodynamic stability of either the intermediates or the final specific complex [95], but substantial desolvation barriers involved in breaking up these interactions in a conformationally restricted protein environment (e.g., Figure 9A) can extend the resident time of the intermediates to allow the rest of the complex to fold with higher efficiency. As demonstrated using a dual-transition state kinetic model [59], efficient folding upon encounter is necessary for achieving facile binding at or near the diffusion-limited basal binding rate, a highly desirable property for signaling and regulatory IDPs that need to constantly evade protein degradation machinery in cell. IDPs are known to be enriched with charges [6]. NCBD and ACTR are no exceptions, with +6 and −8 net charges, respectively (including the flanking loops that remain disordered in the complex [91]). These enriched charges hinder (independent) folding and can protest against aggregation. In addition, long-range electrostatic interactions between these large numbers of complementary charges on NCBD and ACTR could dramatically enhance the encounter rate, similar to electrostatic steering, which is known to be important in interactions of globular protein [111]. Furthermore, the complementary pattern of charge, especially within the predicted mini folding core involving the C-termini (Figure 1), suggests that long-range electrostatic interactions could further promote folding-competent encounter complexes before transiently stabilizing the on-pathway intermediates via formation of short-range salt-bridge network. These effects can enhance the efficiency of folding upon encounter to promote facile recognition.

The current study also reveals important limitations in both the protein force field accuracy and sampling capability, especially for modeling IDPs of moderate sizes and with complex residual structures. These limitations underscore the importance of continual development of the protein force field, with increased focus on balancing various competing interactions to allow an accurate description of not only a few (native) folds but also the whole conformational equilibrium [82], [112]. Sampling methodologies clearly need to improve. The standard temperature REX-MD has failed to achieve convergence for the disordered ensemble of NCBD within 100 ns. Besides limited simulation timescale, certain limitations of the implicit solvent protein force field also contributed. In particular, current empirical protein models have been shown to contain a systematic bias to over-stabilize protein-protein interactions [113], [114]. Furthermore, simple surface area-based estimation of the nonpolar solvation free energy employed in most current implicit solvent models also tends to over-stabilize nonspecific compact protein states [82]. The standard temperature REX-MD clearly has limited ability to sample alternative deeply trapped low energy states with high efficiency. These limitations together have also prevented us from more directly investigating the proposed mechanistic roles of electrostatic interactions using atomistic simulations. Despite these outstanding limitations, the key mechanistic features derived from atomistic physics-based simulations, coarse-grained topology-based modeling, and various biophysical measurements are remarkably consistent, which suggests that an integration of multi-scale modeling and experimentation can provide a viable approach for understanding the functional and control of IDPs.

## Methods

### REX/GBSW simulations of free NCBD and ACTR

Only segments of the NCBD and ACTR domains that are structured in the complex are included in the current simulations, which include residues 2066–2112 for NCBD (in mouse CBP numbering; SALQD LLRTL KSPSS PQQQQ QVLNI LKSNP QLMAA FIKQR_2105_ TAKYV AN) and residues 1040–1086 for the ACTR domain (in human ACTR numbering; E GQSDE RALLD QLHTL LSNTD ATGLE EID_1068_RA LGIPE LVNQG QALEP K). The peptide termini are neutralized using with either acetyl (Ace) or amine (NH2) groups. A previously optimized GBSW/MS2 model was used in all implicit solvent simulations unless otherwise noted [72]. This model adopts an effective approximation of the molecular surface for defining the solute-solvent boundary, which is believed to be more physical compared to the van der Waals-like surface used in the original GBSW model [115], [116]. Importantly, the GBSW/MS2 model has also been carefully optimized to balance solvation and intramolecular interactions and can reasonably capture the competition between α and β secondary structures. Specifically for NCBD/ACTR, the structure of the complex (PDB: 1kbh [91]) remains stable in the GBSW/MS2 force field for over 100 ns, but substantially deviates from the native conformation in the original GBSW protein force field (see Figure S4).

REX was used to enhance the sampling of the accessible conformational space of free NCBD and ACTR. For this, the Multiscale Modeling Tools for Structural Biology (MMTSB) toolset [117] (http://www.mmtsb.org) was used in conjunction with CHARMM [118], [119]. The basic idea of REX is to simulate multiple non-interacting replicas at different temperatures simultaneously. Periodically, one attempts to exchange the simulation temperatures between pairs of replicas based on a Metropolis criterion derived from the detail balance principle. As such, not only the resulting random walk in the temperature space facilitates the system to cross the energy barriers and exploit the conformational space more efficiently, but proper canonical ensembles are also generated at all temperatures, allowing direct calculation of thermodynamic properties for comparison with experiments. We performed two independent REX simulations for each peptide, initiated from the folded structure extracted from the complex (control) and a fully extended conformation (folding), respectively. Comparison of the calculated structure ensembles from these independent control and folding runs with dramatically different initial conditions allows rigorous assessment of the convergence. In each REX simulation, 16 replicas were simulated at temperatures exponentially distributed from 270 to 500 K. SHAKE [120] was applied to fix the lengths of all hydrogen-related bonds, allowing a 2.0 fs molecular dynamics (MD) time step. Temperature exchanges between neighboring replicas were attempted every 2 ps, and the total length of each REX simulation was 100 ns (50,000 REX cycles). Similar REX/GBSW protocols have proven effective in calculating the disordered structural ensembles for other IDPs (albeit of smaller sizes than NCBD and ACTR studied in the current work) [41], [55]. All analysis was performed based on the conformations sampled during the last 60 ns of the control simulation at 305 K (where most existing experimental data were acquired), unless otherwise noted. The orientations of helical segments (1044–1058, 1063–1071, 1072–1080 in ACTR; 2067–2076, 2086–2091, 2095–2110 in NCBD) were calculated using the Chothia-Levitt-Richardson algorithm [121] as implemented in CHARMM. The K-means clustering algorithm as implemented in the MMTSB toolset was used to cluster the calculated disordered ensembles based on mutual Cα RMSD distances. Various clustering radii ranging from 1.5 to 4.5 Å were tested before an optimal radius of 3.0 Å was used for the final clustering results presented. All molecular visualizations were generated using the VMD software [122].

### Room temperature and high-temperature simulations of the wild-type and mutant NCBD/ACTR complexes

The same peptide segments defined above were included the simulations of the complex. The model 1 from the NMR ensemble (PDB: 1kbh) was first equilibrated in the GBSW/MS2 implicit solvent using energy minimization and short MD with weak harmonic positional restraints imposed on all backbone heavy atoms. Subsequently, a 160 ns unrestrained simulation was performed at 300 K to examine the structural stability and dynamics of the complex near its native basin. The native structure of the NCBD:R2105L/ACTR:D1068L double-Leu mutant complex was prepared by computational mutagenesis and then equilibrated using a similar protocol as described above. To identify the optimal temperatures for unbinding/unfolding simulations, a series of pilot simulations was performed at temperatures ranging from 350 K to 500 K (e.g., see Figure S5). At the optimal temperature, the complex should unfold/unbind within tractable time scales (e.g., 10–20 ns) while retaining important details of the unfolding/unbinding pathways. Once such optimal temperatures were chosen (450–475 K for the wild-type and 450 K for the mutant), 50 independent high-temperature simulations of 10–20 ns in length were initiated from the equilibrated native structures with different initial velocities. The results presented in this work are averages computed from 50 unfolding simulations unless otherwise noted. For native fraction analysis, a list of native tertiary contacts (shown in Figure S6) was first identified using the equilibrated native structure based on side chain minimal heavy atom distances with a 4.2 Å cutoff. The native contacts were then divided into inter-molecular and intra-molecular categories. In analysis of the high-temperature simulation trajectories, a contact was considered formed when the minimal heavy atom distance between two side chains was no greater than 4.5 Å. Helicity of various helical segments was calculated based on the hydrogen bonding patterns using the COOR SECS module of CHARMM.

### Explicit solvent high-temperature simulations

Additional high-temperature unfolding and unbinding simulations of the wild-type complex were performed in TIP3P water to examine the unfolding/unbinding pathway and in particular the putative role of the buried salt-bridge between NCBD:R2105 and ACTR:D1068 in (transiently) stabilizing the intermediate state(s). For this, the equilibrated NCBD/ACTR complex was placed in a cubic water box with periodic boundary conditions imposed. The final solvated system contains 9176 TIP3P water molecules and the box size is ∼65 Å. Two potassium ions were added to neutralize the total charge. The proteins were described by the CHARMM22/CMAP protein force field [73]–[76]. The particle mesh Ewald method was used for long-range electrostatic interactions [123], and the van de Waals interactions were smoothly switched off from 12 to 13 Å. Lengths of all hydrogen-related bonds were kept constant with SHAKE [120], and the MD time step was 2 fs. After 10 ps of NPT equilibration at 300 K, a set of 10 independent NVT productions was carried out at 500 K up to 10 ns until the dimensions of the proteins exceed those of the periodic box. The dynamic time step was reduced to 1 fs in the NVT production simulations for numerical stability.

### Free energy calculations

An umbrella sampling protocol [77] was used to compute the PMFs between the side chains of Asp and Arg, either constrained in a head-to-head configuration [77] (see Figure 9) or allowed to freely rotate. In the constrained setup, the side chains were allowed to move only in fixed orientations along the reaction coordinate (indicated by a dashed line in Figure 9), enforced using the MMFP module in CHARMM. For explicit solvent simulations, solutes were solvated by either ∼710 TIP3P waters in a rectangular box (for the constrained PMF) or by ∼1040 TIP3P waters in a truncated octahedral box (for the unconstrained PMF). Periodic boundary conditions were imposed. Non-bonded and other setups are identical to those described above for explicit solvent high-temperature simulations. Harmonic restraint potentials were placed every 0.5 Å along the reaction coordinate with a force constant of 5.0 kcal/mol/Å^2^. For each umbrella-sampling window, the system was first equilibrated for 60 ps, followed by 2 ns (constrained PMF) or 4 ns (unconstrained PMF) NPT production at 300 K and 1 atm. The final PMFs were calculated using the weighted histogram analysis method (WHAM) [124]. The constrained PMF in GBSW/MS2 was computed by direct translation of the side chains along the reaction coordinate, and the unconstrained PMF in GBSW/MS2 was computed in the same umbrella sampling protocol except that implicit solvent was used instead of TIP3P waters. Convergence of the PMFs was examined by comparing results from the first and second halves of the data and was shown to be on the order of 0.2 kcal/mol.

## Supporting Information

Figure S1 **Convergence of the calculated residue helicity of free NCBD.** Residue helicities calculated using different segments of the folding (A) and control (B) REX simulations are shown. Only conformations sampled at 305 K were included in the analysis.
(TIF)

Figure S2 **Additional conformational properties of free NCBD.** A) Distributions of the radius of gyration, and B) Cα RMSF profiles at 305 K, calculated from the last 60 ns of control (red traces) and folding (black traces) simulations.
(TIF)

Figure S3 **Orientations of NCBD Cα_1_ and Cα_3_ with respect to Cα_2_.** Conformations that belong to the six most populated clusters of free NCBD sampled at 305 K are color-coded. See the caption of Figure 3 in the main text for additional information.
(TIF)

Figure S4 **Summary of control simulations of the NCBD/ACTR complex in GBSW and GBSW/MS2.** A) Backbone RMSD as a function of time. B) Number of helical residues as a function of time. C) The Cα RMSF profiles computed from the last 50 ns of the 100 ns control simulations. Helical segments of ACTR and NCBD are marked. D–E) The final snapshots overlaid with the PDB structure (shown in gray cartoon). The results suggest that the NCBD/ACTR complex is unstable in GBSW both at the secondary and tertiary levels. In contrast, the complex remains reasonably stable in GBSW/MS2, with significant fluctuations mainly observed in the C-terminal segment of ACTR, and to a lesser extent in the NCBD C-terminus (see panel C).
(TIF)

Figure S5 **Trial unfolding simulations in GBSW/MS2 at different temperatures.** The numbers of helical residues of NCBD and ACTR are monitored to detect the unbinding/unfolding of the complex.
(TIF)

Figure S6 **Tertiary contacts of the NCBD/ACTR complex.** The contacts were derived based on the first model of PDB:1kbh. Residues are considered in contact if the minimal heavy atom distance is no more than 4.2 Å. The black bars indicate the ranges of all helical segments in NCBD and ACTR. Although there are a large number of intermolecular contacts (62; black dots), there are only 11 (blue dots) and 19 (red dots) tertiary intramolecular contacts for ACTR and NCBD, respectively.
(TIF)

Figure S7 **Evolution of various contact fractions in GBSW/MS2 simulations at 450 and 475 K.** The grey traces were calculated from averaging 50 independent simulations at corresponding temperatures, and the colored traces are 50-ps running averages.
(TIF)

Figure S8 **Distances between key charged residues during three representative unfolding simulations at 475 K.** For Arg and Glu/Asp pairs, the distance between the side chain carbonyl carbon and Arg C_Z_ distance is shown. For Lys and Glu/Asp pairs, the distance between the side chain carbonyl carbon and amide nitrogen is shown.
(TIF)

Figure S9 **Evolution of various contact fractions during unfolding simulations in TIP3P at 500 K.** All curves were calculated from averaging 10 independent simulations of 3 to 4 ns in length (only the first 3 ns are shown). The grey traces were calculated from averaging 50 independent simulations, and the colored traces are 50-ps running averages. The results are consistent with key observations derived from GBSW/MS2 simulations. Specifically, 1) the baseline mechanism for coupled binding and folding of NCBD is an induced folding-like one, where binding precedes folding (Panel A); Specifically, fitting of *Q*_inter_ and *Q*_NCBD_ traces to single exponential functions yields half times, τ = 0.35 ns and 0.25 ns, respectively. 2) The C-terminal segments initiate binding (thus the first helices unbind the first; see black traces in Panels C–D); 3) the local native and non-native salt-bridges persist in the partially unfolded and partially unbound intermediate state (Panel B). Note that the helical secondary structures appear to be over-stabilized (e.g., see the blue trace in Panel A), which is a known artifact of the current version CHARMM22/CMAP explicit solvent force field.
(TIF)

Figure S10 **Evolution of various contact fractions during unfolding simulations of the mutant NCBD/ACTR complex at 450 K.** The grey traces were calculated from averaging 50 independent simulations, and the colored traces are 50-ps running averages. The simulations were 15 ns in length. The complex unfolds rapidly and thus only results from the first 5 ns are shown.
(TIF)
