# Supplementary figures and images for: Residual Structures, Conformational Fluctuations, and Electrostatic Interactions in the Synergistic Folding of Two Intrinsically Disordered Proteins
Source: PLoS Comput Biol. 2012 Jan 12;8(1):e1002353. doi: 10.1371/journal.pcbi.1002353 (PMC3257294; doi:10.1371/journal.pcbi.1002353)

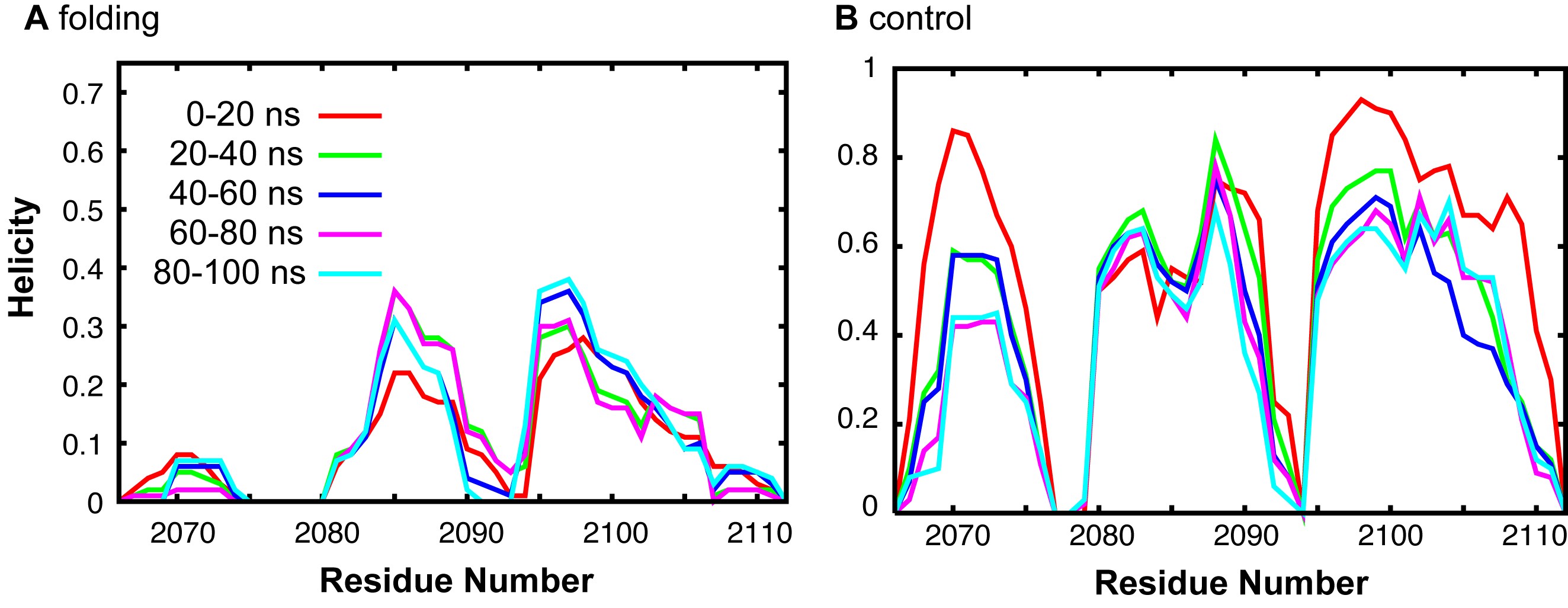

Supplement: Figure S1 — Convergence of the calculated residue helicity of free NCBD. Residue helicities calculated using different segments of the folding (A) and control (B) REX simulations are shown. Only conformations sampled at 305 K were included in the analysis. (TIF) [file pcbi.1002353.s001.tif]

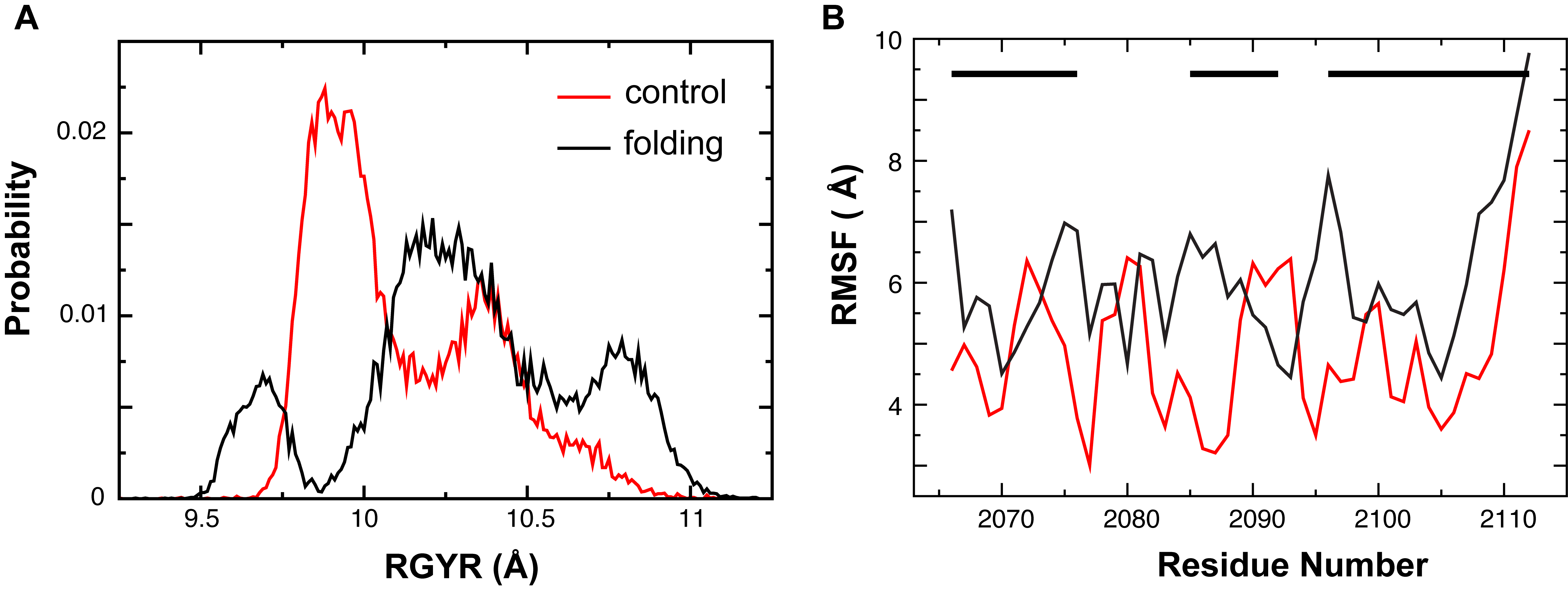

Supplement: Figure S2 — Additional conformational properties of free NCBD. A) Distributions of the radius of gyration, and B) Cα RMSF profiles at 305 K, calculated from the last 60 ns of control (red traces) and folding (black traces) simulations. (TIF) [file pcbi.1002353.s002.tif]

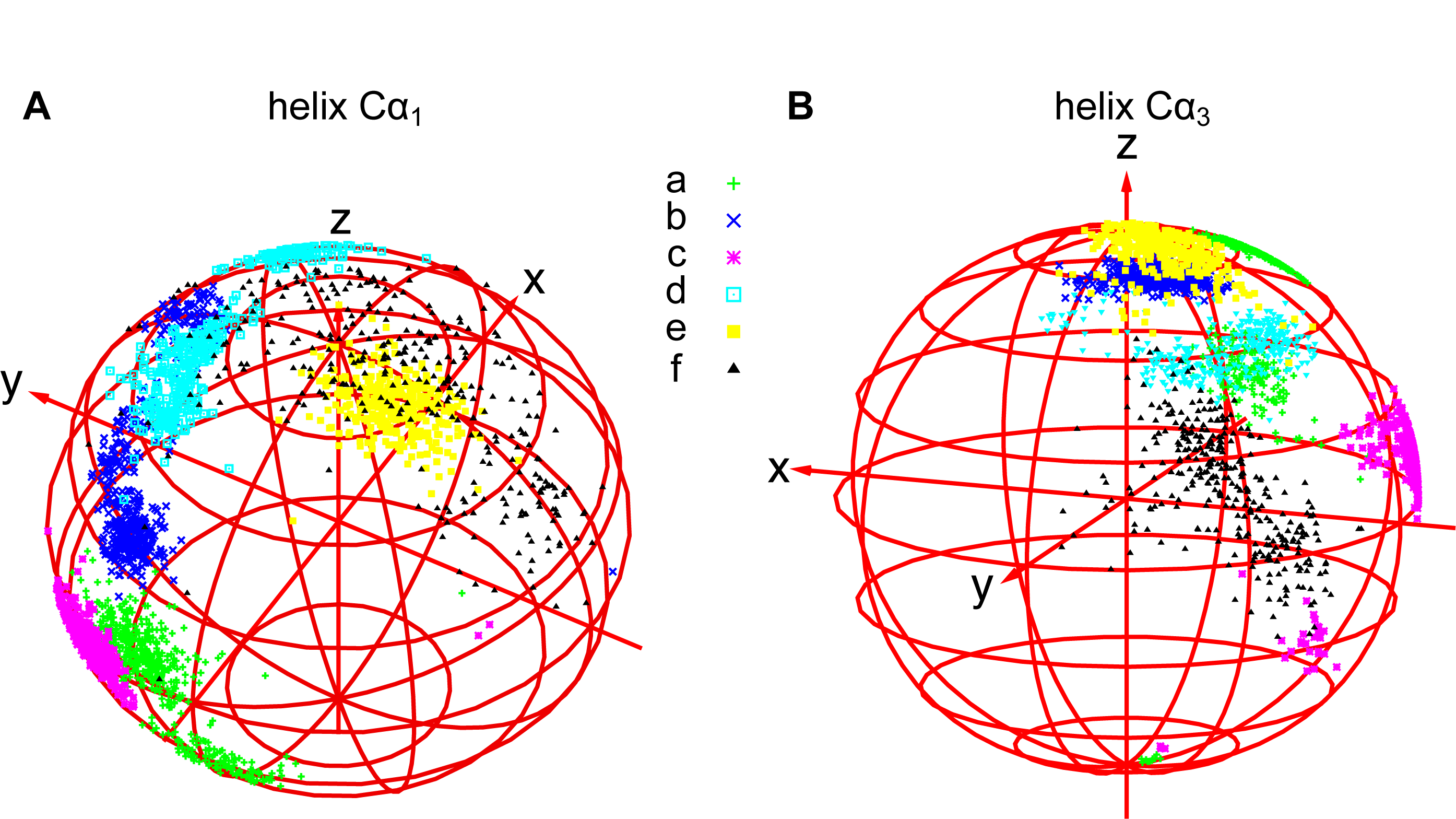

Supplement: Figure S3 — Orientations of NCBD Cα1 and Cα3 with respect to Cα2. Conformations that belong to the six most populated clusters of free NCBD sampled at 305 K are color-coded. See the caption of Figure 3 in the main text for additional information. (TIF) [file pcbi.1002353.s003.tif]

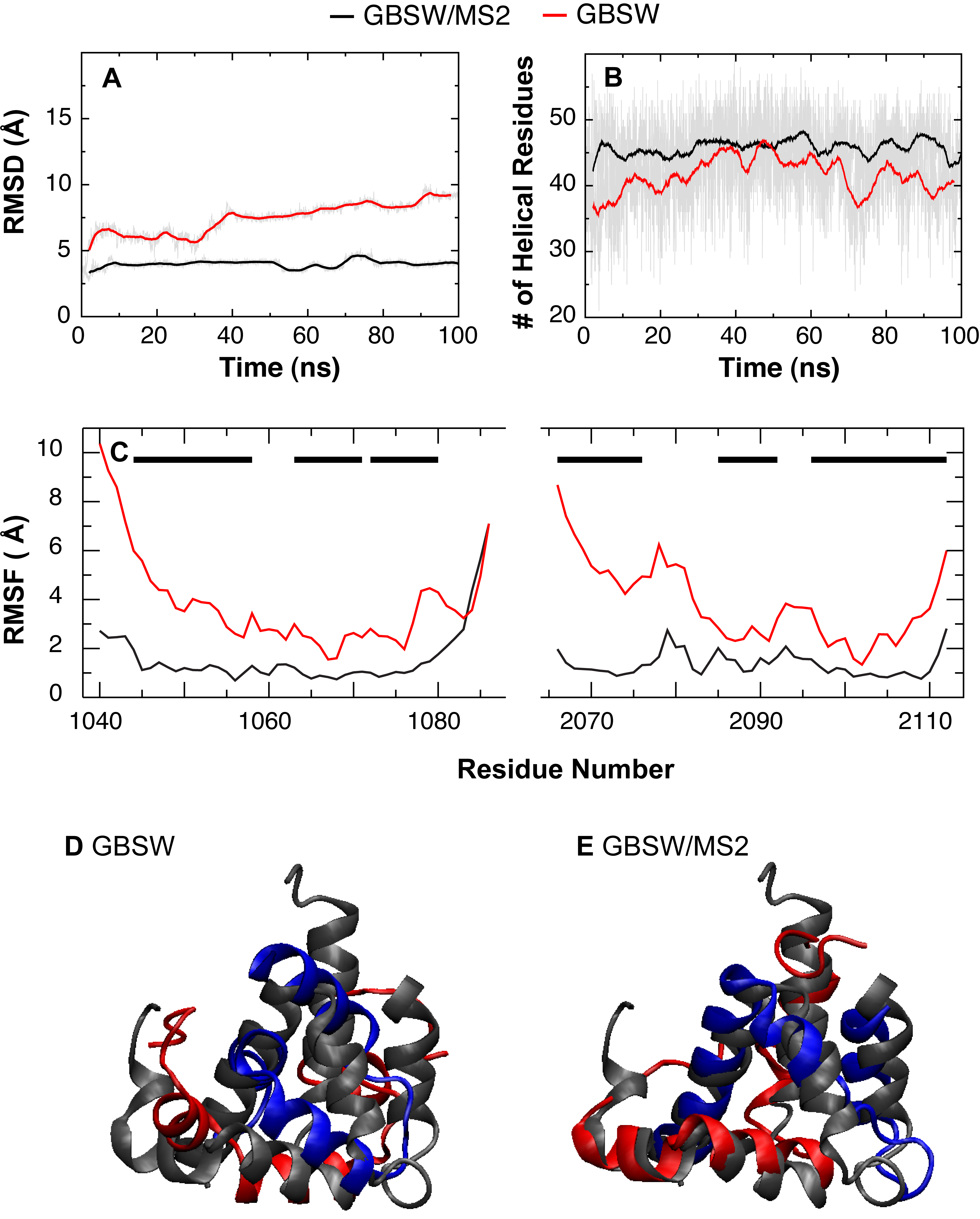

Supplement: Figure S4 — Summary of control simulations of the NCBD/ACTR complex in GBSW and GBSW/MS2. A) Backbone RMSD as a function of time. B) Number of helical residues as a function of time. C) The Cα RMSF profiles computed from the last 50 ns of the 100 ns control simulations. Helical segments of ACTR and NCBD are marked. D–E) The final snapshots overlaid with the PDB structure (shown in gray cartoon). The results suggest that the NCBD/ACTR complex is unstable in GBSW both at the secondary and tertiary levels. In contrast, the complex remains reasonably stable in GBSW/MS2, with significant fluctuations mainly observed in the C-terminal segment of ACTR, and to a lesser extent in the NCBD C-terminus (see panel C). (TIF) [file pcbi.1002353.s004.tif]

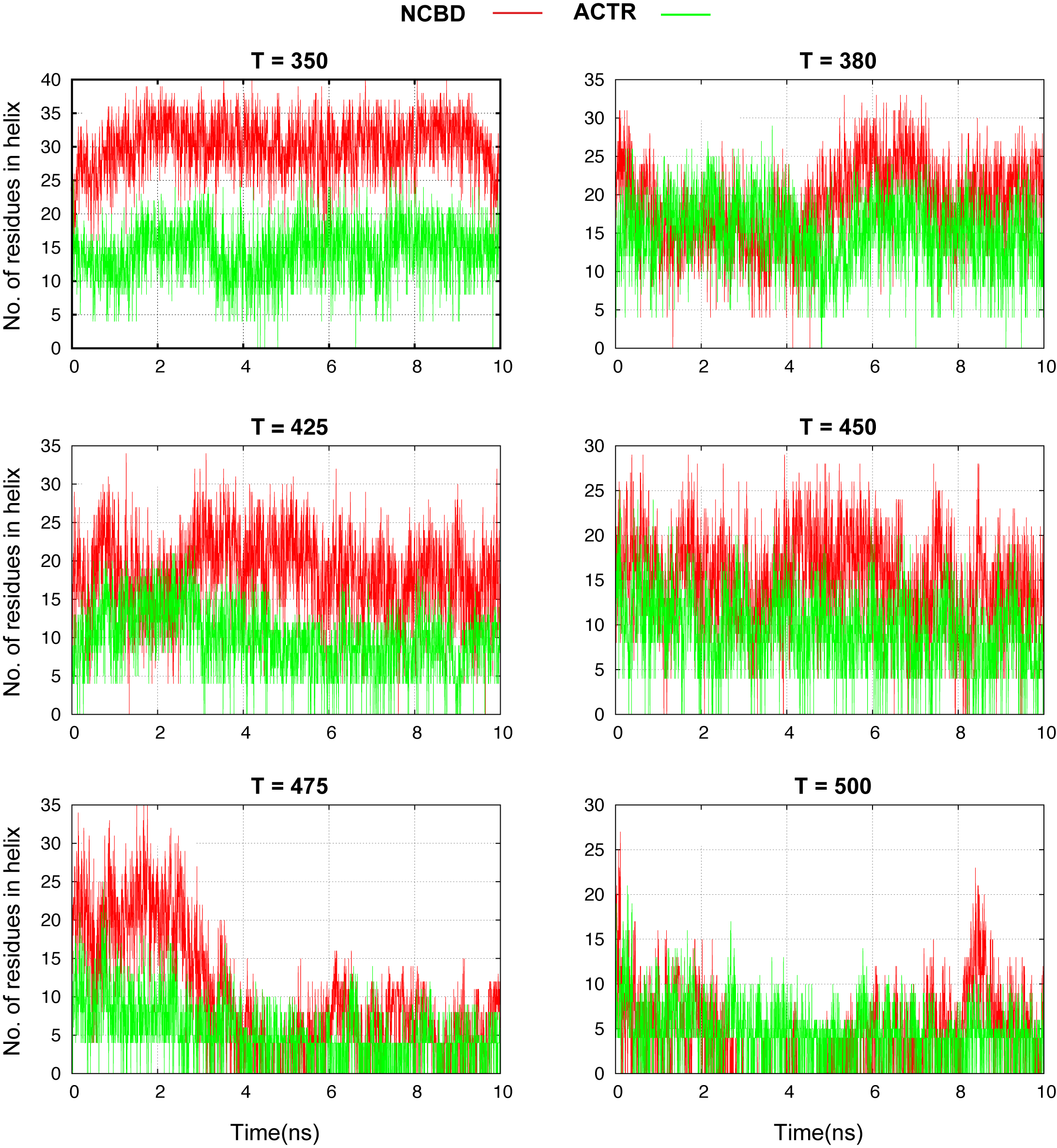

Supplement: Figure S5 — Trial unfolding simulations in GBSW/MS2 at different temperatures. The numbers of helical residues of NCBD and ACTR are monitored to detect the unbinding/unfolding of the complex. (TIF) [file pcbi.1002353.s005.tif]

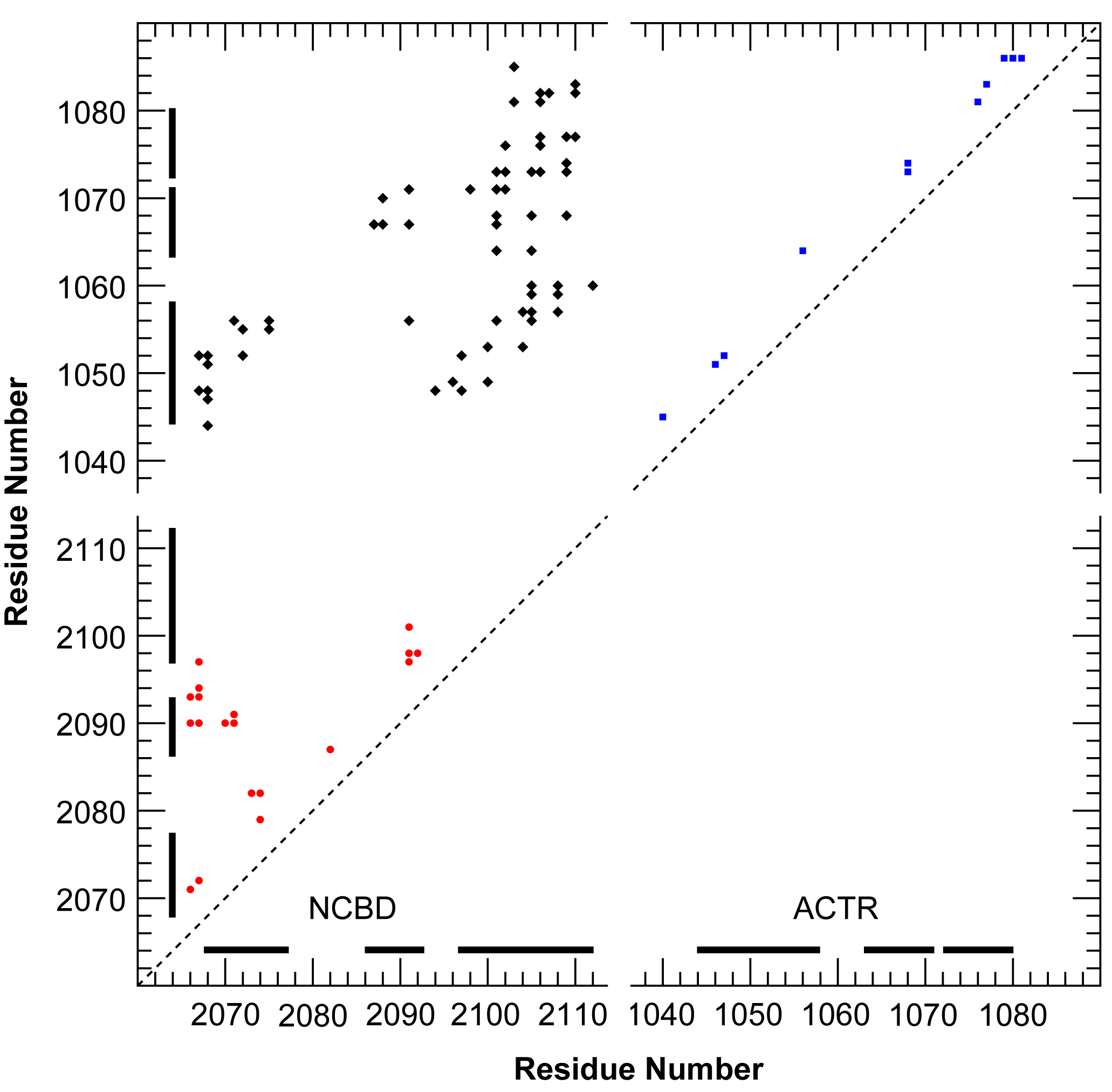

Supplement: Figure S6 — Tertiary contacts of the NCBD/ACTR complex. The contacts were derived based on the first model of PDB:1kbh. Residues are considered in contact if the minimal heavy atom distance is no more than 4.2 Å. The black bars indicate the ranges of all helical segments in NCBD and ACTR. Although there are a large number of intermolecular contacts (62; black dots), there are only 11 (blue dots) and 19 (red dots) tertiary intramolecular contacts for ACTR and NCBD, respectively. (TIF) [file pcbi.1002353.s006.tif]

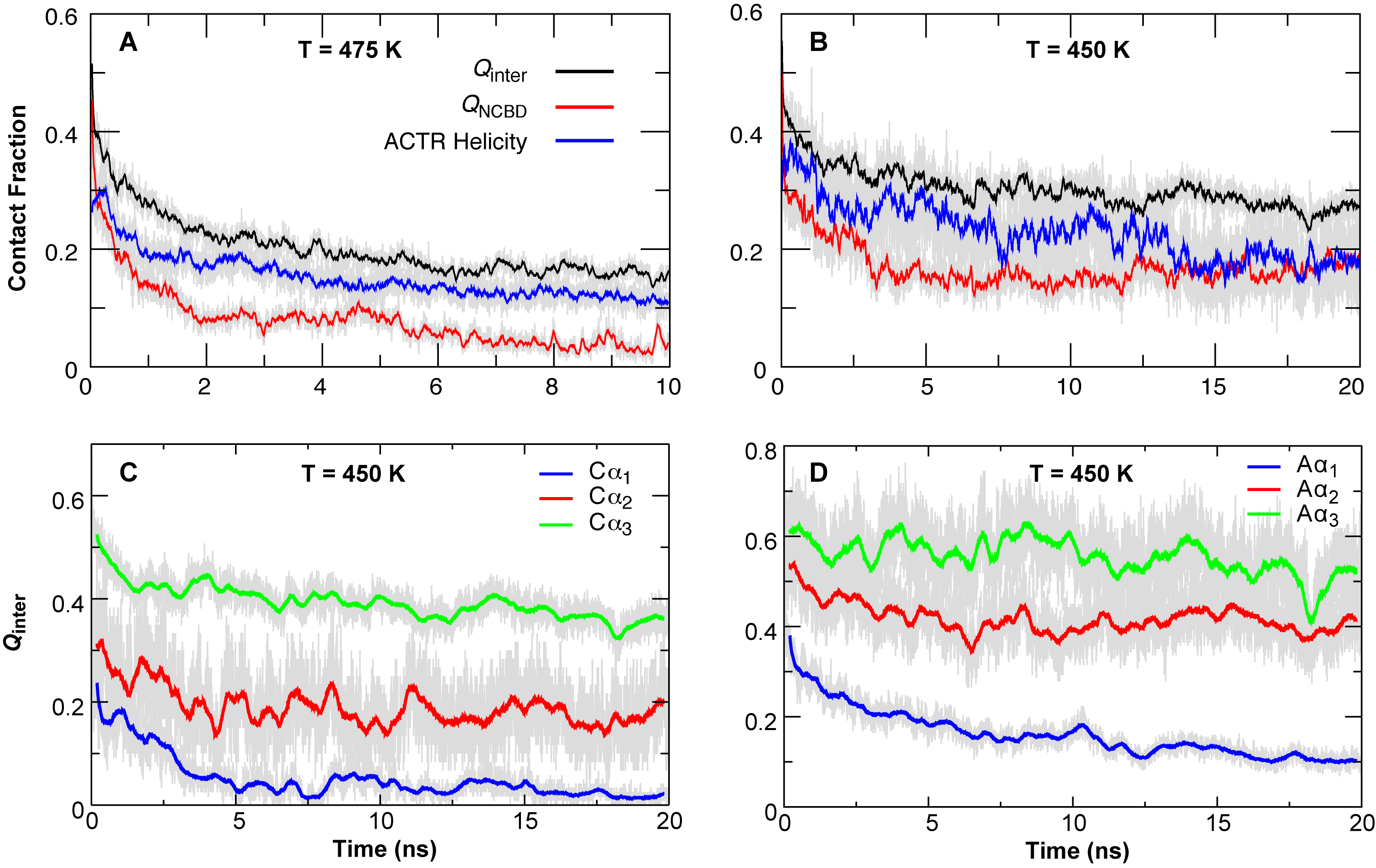

Supplement: Figure S7 — Evolution of various contact fractions in GBSW/MS2 simulations at 450 and 475 K. The grey traces were calculated from averaging 50 independent simulations at corresponding temperatures, and the colored traces are 50-ps running averages. (TIF) [file pcbi.1002353.s007.tif]

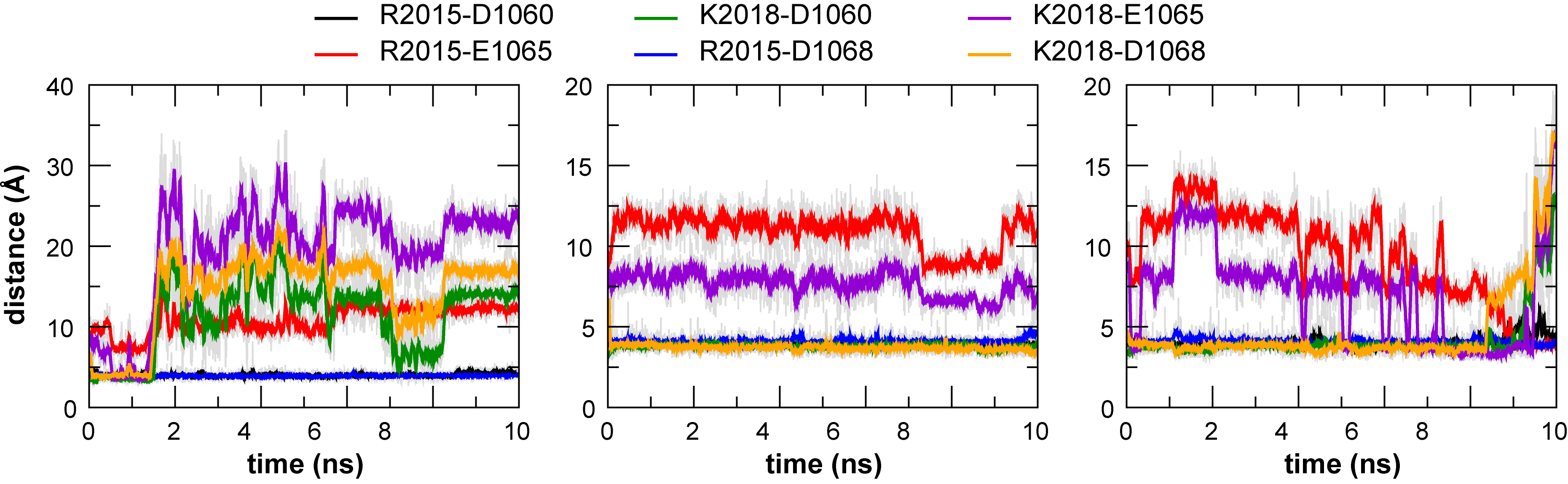

Supplement: Figure S8 — Distances between key charged residues during three representative unfolding simulations at 475 K. For Arg and Glu/Asp pairs, the distance between the side chain carbonyl carbon and Arg CZ distance is shown. For Lys and Glu/Asp pairs, the distance between the side chain carbonyl carbon and amide nitrogen is shown. (TIF) [file pcbi.1002353.s008.tif]

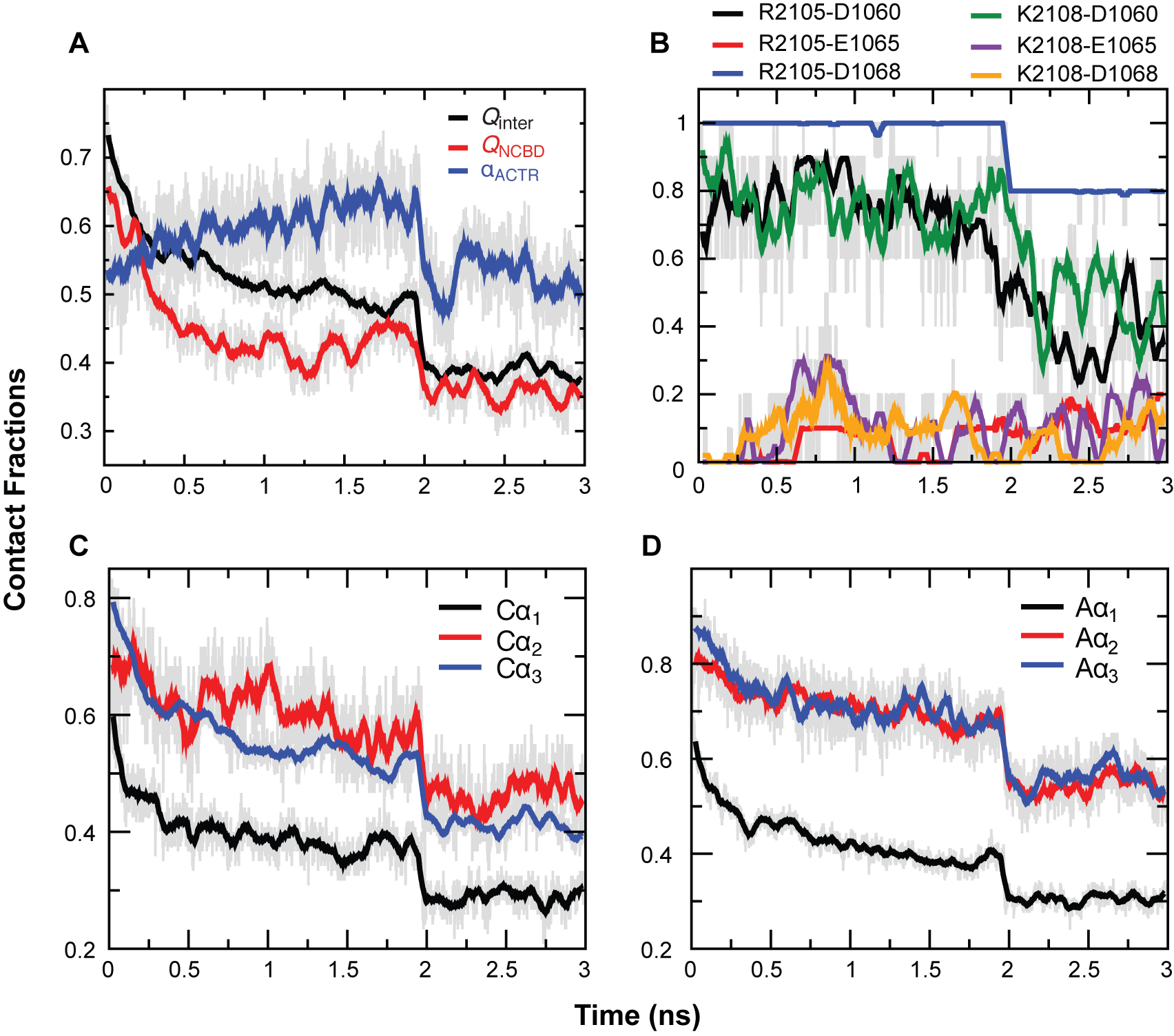

Supplement: Figure S9 — Evolution of various contact fractions during unfolding simulations in TIP3P at 500 K. All curves were calculated from averaging 10 independent simulations of 3 to 4 ns in length (only the first 3 ns are shown). The grey traces were calculated from averaging 50 independent simulations, and the colored traces are 50-ps running averages. The results are consistent with key observations derived from GBSW/MS2 simulations. Specifically, 1) the baseline mechanism for coupled binding and folding of NCBD is an induced folding-like one, where binding precedes folding (Panel A); Specifically, fitting of Q inter and Q NCBD traces to single exponential functions yields half times, τ = 0.35 ns and 0.25 ns, respectively. 2) The C-terminal segments initiate binding (thus the first helices unbind the first; see black traces in Panels C–D); 3) the local native and non-native salt-bridges persist in the partially unfolded and partially unbound intermediate state (Panel B). Note that the helical secondary structures appear to be over-stabilized (e.g., see the blue trace in Panel A), which is a known artifact of the current version CHARMM22/CMAP explicit solvent force field. (TIF) [file pcbi.1002353.s009.tif]

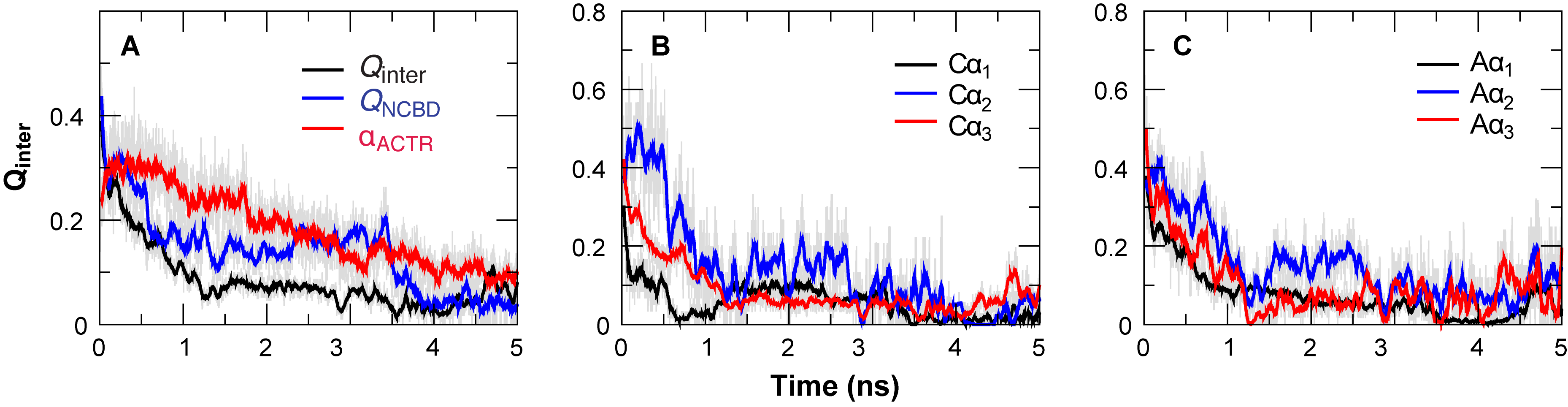

Supplement: Figure S10 — Evolution of various contact fractions during unfolding simulations of the mutant NCBD/ACTR complex at 450 K. The grey traces were calculated from averaging 50 independent simulations, and the colored traces are 50-ps running averages. The simulations were 15 ns in length. The complex unfolds rapidly and thus only results from the first 5 ns are shown. (TIF) [file pcbi.1002353.s010.tif]
